# Unveiling the mechanism of lattice-mismatched crystal growth of a core–shell metal–organic framework†
†Electronic supplementary information (ESI) available. Experimental details, preliminary characterisation results, additional AFM micrographs and Raman spectra. See DOI: 10.1039/c9sc03131f


**DOI:** 10.1039/c9sc03131f

**Published:** 2019-08-27

**Authors:** Fajar I. Pambudi, Michael W. Anderson, Martin P. Attfield

**Affiliations:** a Department of Chemistry , The University of Manchester , Manchester , M13 9PL , UK . Email: m.attfield@manchester.ac.uk; b Department of Chemistry , Universitas Gadjah Mada , Sekip Utara , Yogyakarta , 55281 , Indonesia

## Abstract

Determining the effect of severe lattice mismatch on the crystal growth mechanism and form of epitaxially grown materials is vital to understand and direct the form and function of such materials. Herein, we report the use of atomic force microscopy to reveal the growth of a shell metal–organic framework (MOF) on all faces of a core MOF that has similar *a*, *b*-lattice parameters but a ∼32% mismatch in the *c*-lattice parameter. The work shows the mechanism through which the shell MOF overcomes the core terrace height mismatch depends on that mismatch being reduced before overgrowth of continuous shell layers can occur. This reduction is achieved *via* a process of growth of non-continuous shell layers that are terminated by terrace edges of the core. The crystal form of the shell MOF is heavily influenced by the lattice mismatch which hinders continuous spreading of the interfacial and subsequent shell layers on some facets. The results exemplify the crystal growth versatility of MOFs to accommodate large lattice mismatch, to house many more functional defects in a core–shell MOF than either of the component MOFs, and has broader implications for engineering lattice-mismatched core–shell materials in general.

## Abstract 2

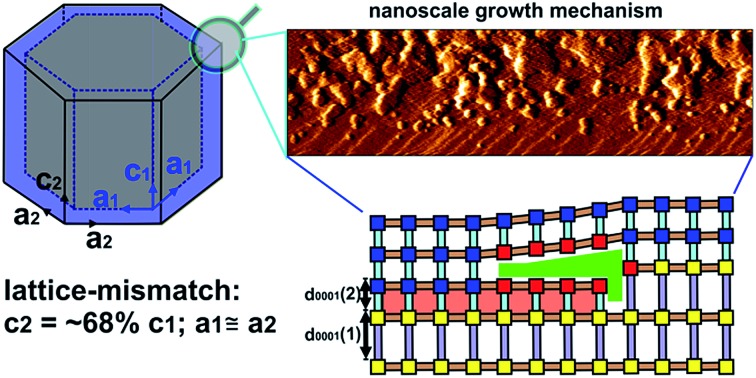
Real time microscopy reveals the nanoscopic epitaxial growth mechanism and form of a severely lattice-mismatched shell MOF in a core–shell MOF.

## Introduction

Porous metal–organic frameworks, or coordination polymers, form the largest family of crystalline porous material that is commanding great interest due to their diverse array of form and function.^1^ Both pure component MOFs^2^–^5^ and mixed component MOFs^6^–^10^ have been synthesized. One class of the latter that is still somewhat rare, but accesses additional levels of structural and functional diversity, is core–shell lattice-mismatched MOFs (CS-LM-MOFs) in which the shell MOF has a different framework composition and unit cell from the underlying core MOF to which it is directly connected.^11^–^18^ Currently, information is known concerning the crystal morphology and composition of these CS-LM-MOFs, and the diffraction details of the shell MOF and its crystallographic orientation relative to the core.^11^–^17^ However, little is known concerning the actual nanoscale growth mechanism of the lattice-mismatched shell MOF over the core MOF, and the influence on the crystal form of the shell MOF resulting from such mismatch. Such a dearth of knowledge is surprising given that the exciting properties and functionalities of CS-LM-MOFs, such as size selective catalysis^14^ and orientation dependent plasmon resonance,^15^ may critically depend on the overall form of the MOF shell, the defects it contains and its interfacial structure with the core MOF. *In situ* atomic force microscopy (AFM),^18^,^19^ and the synthesis components and conditions of MOF crystallisation^20^ form an ideal combination for filling this knowledge void^21^–^28^ and providing unprecedented understanding of the formation of CS-LM-MOFs, and lattice-mismatched core–shell materials in general.

**Fig. 1:**
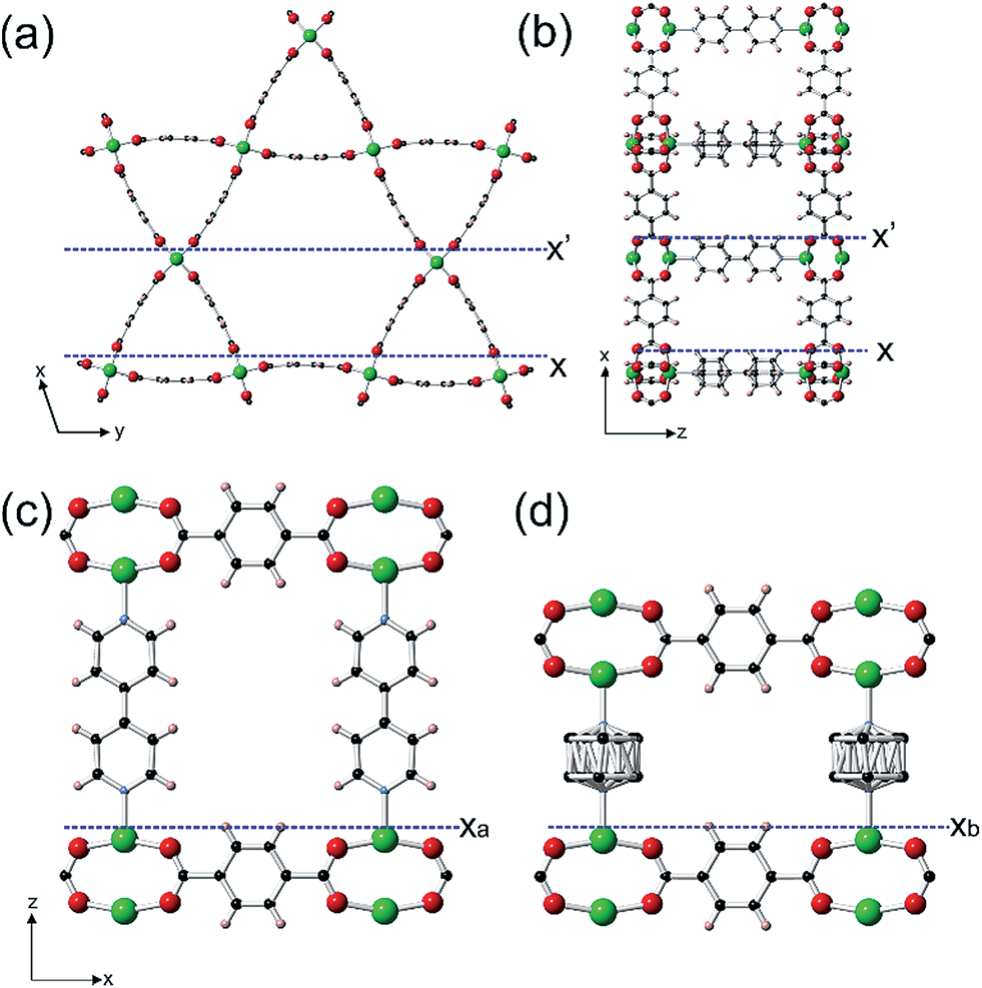
Structure of the Kagome net layer of **1** viewed along a viewed along a 〈0001〉direction (a), the structure of 0001 viewed along a 〈0001〉direction (a), the structure of direction (a), the structure of **1** perpendicular to a {101[combining macron]0} facet viewed along a perpendicular to a {101̄0} facet viewed along a 〈011̄0〉 direction (b), the structure of 011[combining macron]0 perpendicular to a {101̄0} facet viewed along a 〈011̄0〉 direction (b), the structure of direction (b), the structure of **1** perpendicular to a {0001} facet viewed along a perpendicular to a {0001} facet viewed along a 〈011̄0〉 direction (c) and the structure of 011[combining macron]0 perpendicular to a {0001} facet viewed along a 〈011̄0〉 direction (c) and the structure of direction (c) and the structure of **2** perpendicular to a {0001} facet viewed along a perpendicular to a {0001} facet viewed along a 〈011̄0〉 direction (d). The structure of 011[combining macron]0 perpendicular to a {0001} facet viewed along a 〈011̄0〉 direction (d). The structure of direction (d). The structure of **2** is similar to that shown in (a) and (b) except that the bpy ligands are replaced by dabco ligands. The structures are represented in ball-and-stick mode: green: Zn, red: O, light blue: N, black: C, pink: H.

In this work, the construction of a CS-LM-MOF from two isoreticular pillared Kagome net MOFs, [Zn_2_(bdc)_2_(bpy)] (**1**) (*a* = 21.619(8) Å, *c* = 14.104(5) Å)^29^ (bdc = 1,4-benzenedicarboxylate, bpy = 4,4′-bipyridine) and [Zn_2_(bdc)_2_(dabco)] (**2**) (*a* = 21.620(1) Å, *c* = 9.6282(8) Å)^30^ (dabco = diazabicyclo[2,2,2]octane) is followed. MOFs **1** and **2** have similar *a*-lattice parameters but a ∼32% mismatch in *c*-lattice parameters [{*c*(**1**)-*c*(**2**)}/*c*(**1**)] and were selected as the core and shell MOF respectively. Both MOFs are constructed from Zn_2_ paddle wheel dimers units connected by four bdc^2–^ ligands to form Kagome net layers in the {0001} planes pillared by bitopic N-containing linkers in the ligands to form Kagome net layers in the {0001} planes pillared by bitopic N-containing linkers in the 〈0001〉 directions as shown in 0001 ligands to form Kagome net layers in the {0001} planes pillared by bitopic N-containing linkers in the 〈0001〉 directions as shown in directions as shown in Fig. 1. We determine, for the first time, the crystal form of shell MOF and the mechanism by which it is able to grow on a core MOF for which there is a large mismatch in lattice parameter between the core and shell. Such understanding of the formation of CS-LM-MOFs will direct the future design and synthesis of such complex MOF forms with particular regard to the engineering of the interfacial defects.

## Results and discussion

Easily identifiable hexagonal prismatic crystals of **1** were grown as a component of a mixed phase sample (see Fig. S1†) with clearly expressed {0001} and {101[combining macron]0} facets as shown in Fig. S2.† Initial *in situ* AFM studies of the dissolution and crystal growth of **1** under a low supersaturation supernatant of **1** revealed that both the {0001} and {101[combining macron]0} facets were terminated by stable extended growth steps of height 1.4 ± 0.1 nm and 1.9 ± 0.1 nm corresponding to the *d*_0001_ and *d*_101[combining macron]0_ spacing of 1.41 nm and 1.87 nm respectively (see Fig. S3 and S4†). By comparison of cross-sectional analyses derived heights of various surface nuclei and sub-steps with interatomic distances determined from the crystal structure of **1** (see Fig. S3 and S4†), these studies also determined that the terminating surface of the {0001} facets consists of the H atoms of the bdc^2–^ ligands and one Zn atom from each Zn_2_ dimer and that of the {101[combining macron]0} facets consist of the H atoms of the bpy ligands and two Zn atoms from each Zn_2_ dimer as marked by *X*_a_, *X*′ and *X* respectively in Fig. 1 and S5.† These observations are similar to the results reported for other *in situ* crystal growth studies of MOFs where incompletely framework ligated metal species form the terminating surface.^21^–^23^,^26^

**Fig. 2:**
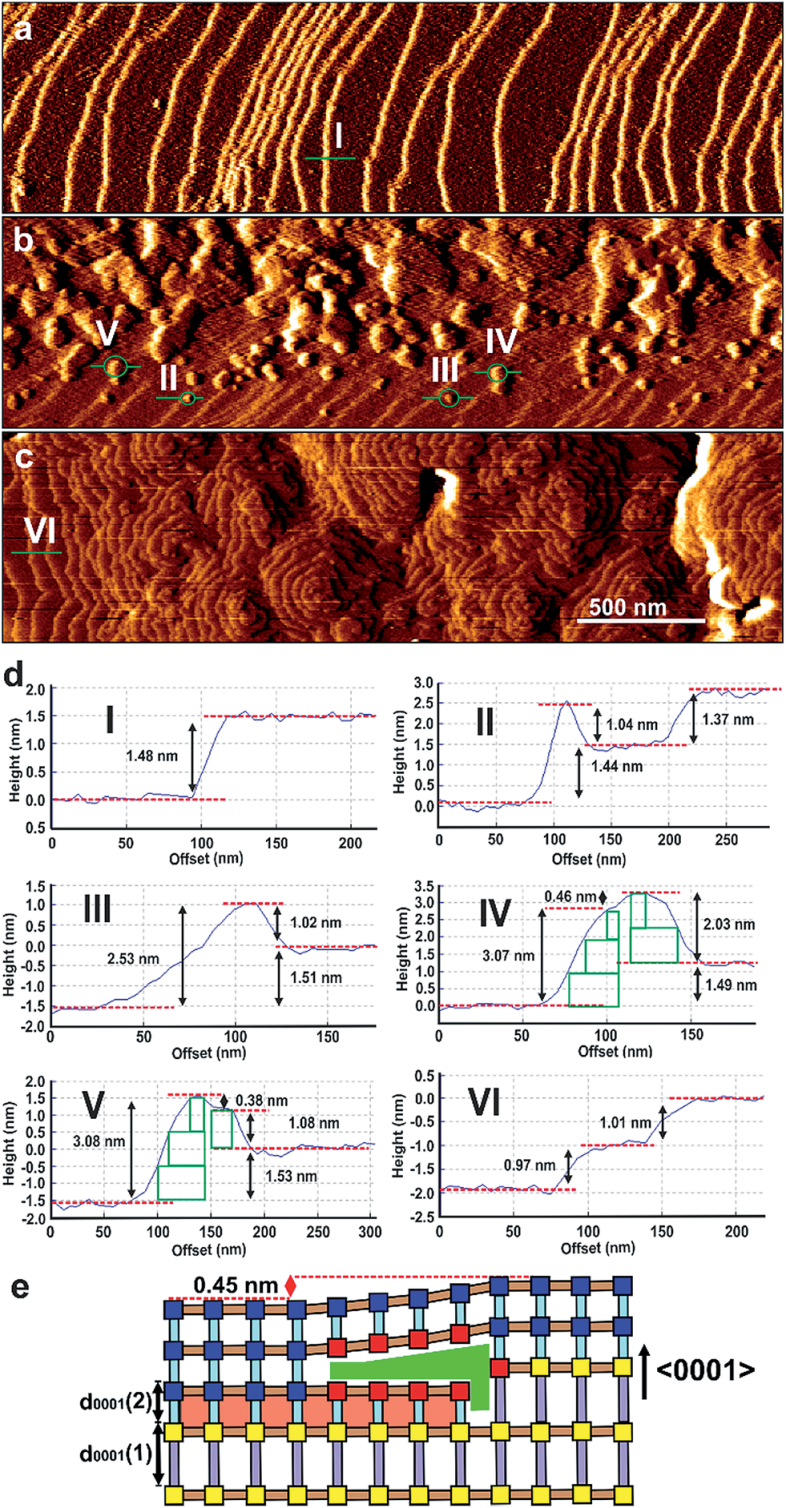
AFM deflection images of a {0001} facet of a crystal of **1** under DMF (a), and 2 min (b) and 8.5 min (c) after injecting a growth solution of **2**. Cross-sectional analyses (d) of the terraces and 2D nuclei marked in (a)–(c). Schematic (e) of the developing nuclei in (d) IV showing overgrowth of a *d*_0001_ terrace of **1** by *d*_0001_ layers of **2**. AFM image size is 3 × 0.8 μm^2^ and covers the same area of the crystal face. Scale bars for (a)–(c) are identical. Key for (e): blue squares – fully coordinated Zn_2_ dimers; red squares – partially coordinated Zn_2_ dimers; grey rods – bpy; pale blue rods – dabco; light brown rods – bdc^2–^.

*In situ* growth of a shell of **2** on a {0001} face of **1** was initially studied for which there is near perfect lattice/structure matching in the *ab*-plane but a potential ∼32% mismatch between the height of a terrace of **1** and a terrace of **2** that will influence the mechanism of shell growth on this face. The *in situ* growth of a shell of **2** was observed on a {0001} face of **1** by replacing the pure DMF over **1** in the *in situ* AFM cell by a slow ingression of a low supersaturation growth solution of **2**. The *in situ* growth images demonstrate the extremely rapid growth of **2** over **1** as shown in Fig. 2, S6 and S7.† Fig. 2a and d(I) shows the surface of **1** under DMF consisting of 1.4 nm high crystal terraces. The subsequent image (Fig. 2b) after injecting the growth solution of **2** features the crystal terraces of **1** in the lower part of the image (Fig. 2b and d(II)), several developing 2D nuclei of **2** on these terraces (Fig. 2b and d(II–V)) and growth islands and terraces of **2** in the upper part of the image. Subsequent images show that the whole surface is covered by growth terraces, hillocks and spirals of **2** with 1.0 nm high crystal terraces as shown in Fig. 2c and d(VI) and represented structurally in Fig. 1d and S5c.† Cross sectional analyses of the 2D nuclei provide crucial insight into the growth mechanism of **2** on **1**. Nuclei of **2** are seen to nucleate at the step edge and on the terrace of **1** with a greater probability of forming near the step edge. These nuclei further develop by a birth-and-spread process of metastable sublayers of dabco and {Zn_2_(bdc)_4_}_*n*_ (see Fig. S6c(III)† and 2d(II) and (III)) to form the 1.0 nm high nuclei of **2**. Also particularly noticeable in Fig. 2b is the nuclei of **2** that have developed over the 1.4 nm high terrace steps of **1** (see Fig. 2d(IV and V)). This *in situ* image provides evidence that individual crystal layers of **2** cannot directly overgrow the terraces of **1** when there is a height mismatch of 1.4 nm but can overgrow the terraces of **1** when the height mismatch is reduced to 0.45 nm through formation of a non-overgrowing layer of **2** that is terminated when it reaches the 1.4 nm high terrace of **1** as schematically represented in Fig. 2e. Subsequent layers of **2** can grow over these 0.45 nm step height mismatches to allow continuous layer spreading of **2** over the {0001} faces of **1**. The nuclei shown in Fig. 2d(IV) and (V) demonstrate that the layers of **2** can overgrow terrace height mismatches of 0.45 nm with movement up (Fig. 2d(IV)) or down (Fig. 2d(V)) and over such a height mismatch. For the latter this requires an additional non-overgrowing layer of **2** to grow that is terminated when it reaches the 1.4 nm high terrace of **1** and provides some indication that the rate of nucleation of a nucleus of **2** on **2** is similar to the rate of terrace overgrowth across the height mismatch.

**Fig. 3:**
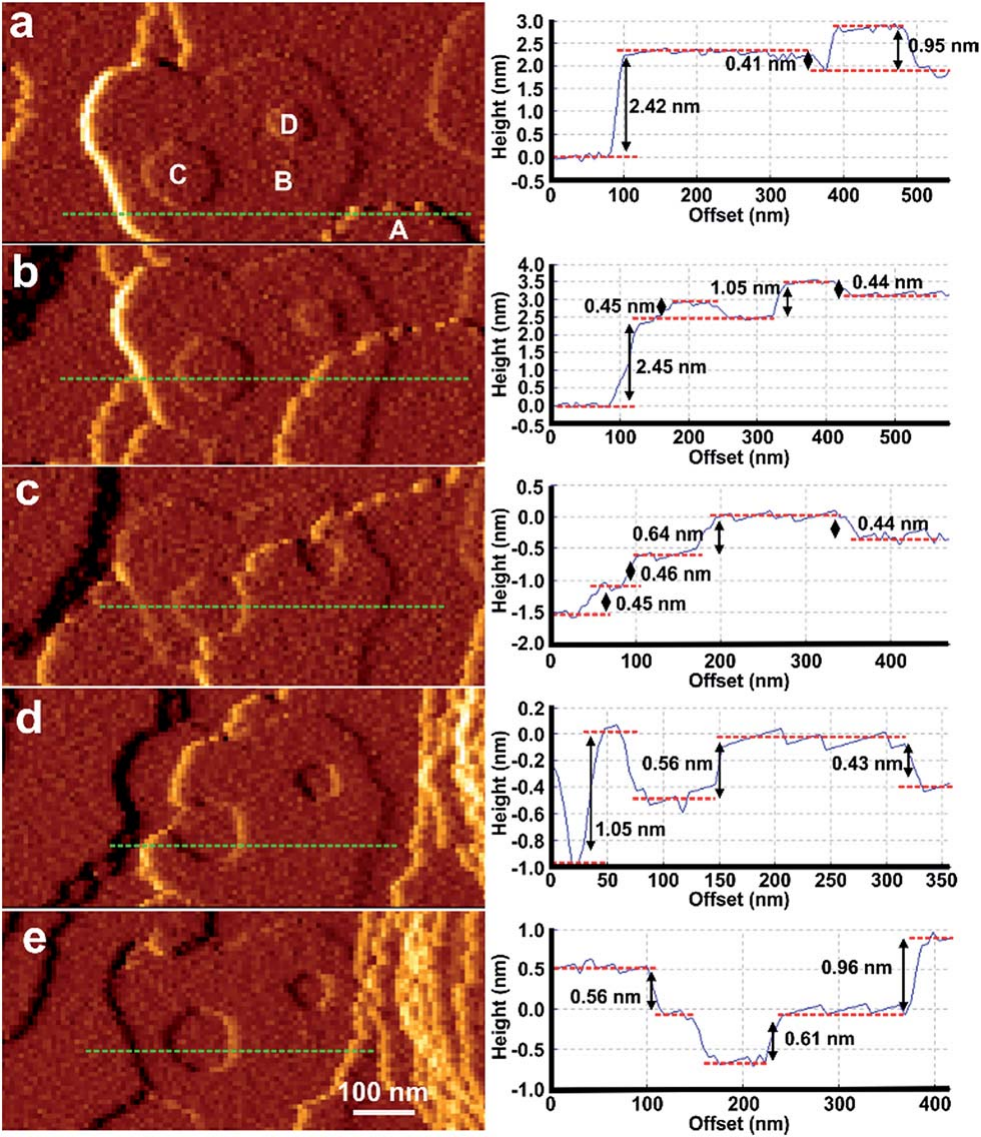
A series of AFM deflection images with associated cross-sectional analyses showing a *d*_0001_-spacing monolayer of **2** (A) overgrowing a large 0.45 nm high island of **2** (B) and growing around smaller 0.45 nm high islands (C and D). AFM images size is 0.7 × 0.4 μm^2^ and scale bars for (a)–(e) are identical.

These 0.45 nm terrace height mismatches are found to persist within the shell of **2** as it grows in thickness as displayed by the 0.45 nm high growth islands in Fig. 3, S8 and S9† that display the *in situ* growth of **2** over **1** monitored for a much longer time than that shown in Fig. 2. Fig. 3, S8 and S9† also demonstrate that layers of **2** can completely overgrow these 0.45 nm high terrace mismatched features corroborating the mechanism of growth proposed for the growing nuclei of **2** over the 0.45 nm height mismatches. Also evidenced in Fig. 3 is the presence of 0.45 nm high mismatched hexagonal growth islands (marked C and D in Fig. 3) around which the developing 1.0 nm layer of **2** cannot overgrow. The approximately 30° rotation of these hexagonal growth islands with respect to the bulk crystal orientation shown in Fig. S10† suggests the existence of domains of **2** or **1** that are structurally misaligned in the (0001) plane with that of the new layer of **2** that may try but is prevented from overgrowing it. This type of defect has not been reported for a MOF but is known to occur on a much smaller scale in hexagonal nanoporous materials such as zeolite L.^31^

The growth mechanism of **2** introduces many framework Zn atoms that will be undercoordinated by the framework organic linkers at the locations of the 0.45 nm terrace height mismatches at the interface of **1** and **2** as represented by the red Zn_2_ nodes in Fig. 2e.

**Fig. 4:**
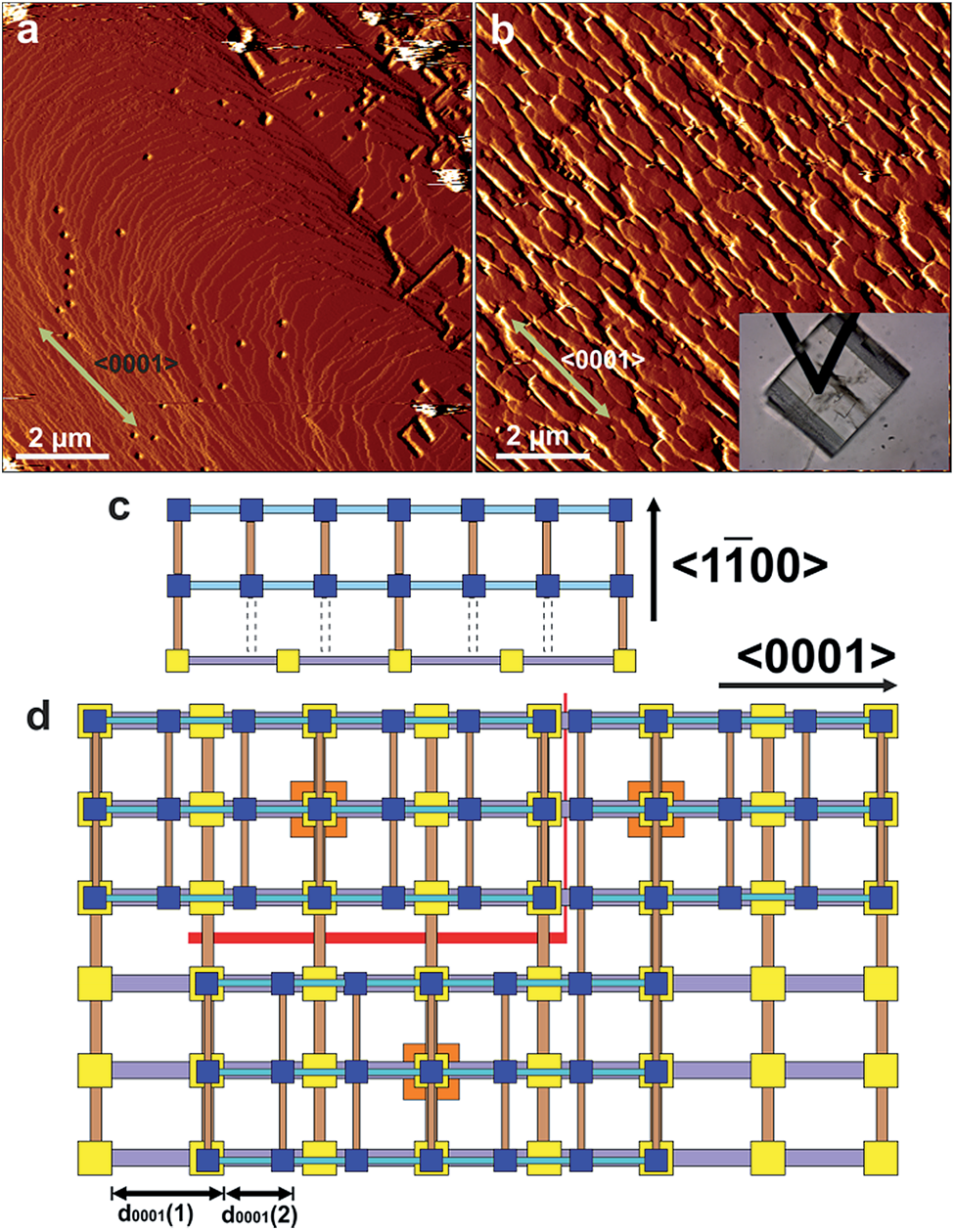
AFM deflection images of a {101[combining macron]0} face of a crystal of **1** under DMF (a) and 240 min (b) after injecting a growth solution of **2**. The inset in (b) is the optical micrograph of the AFM tip and the crystal of **1** allowing comparison of the relative alignment of the growth islands with the bulk crystal orientation. Schematics (c) and (d) of interfacial growth islands of **2** containing under-coordinated Zn_2_ dimer nodes growing on a {101[combining macron]0} face of **1** showing the various point and line defects that can be formed during crystallisation. AFM image size is 10 × 10 μm^2^ and covers the same area of the crystal face. Key for (c) and (d): yellow squares – Zn_2_ dimers of **1**; blue squares – Zn_2_ dimers of **2**; orange squares – nucleating Zn_2_ dimers for growth islands of **2**; grey rods – bpy; pale blue rods – dabco; light brown rods – bdc^2–^; red lines – line defects; grey dotted lines – missing bdc^2–^ linkers.

Attempts at *in situ* growth of a shell of **2** on a {101[combining macron]0}face of **1** were expected to be unsuccessful due to the ∼32% mismatch of the *c*-parameters and thus lattice/structure mismatching of **1** and **2** in the *ac*-plane which has resulted in anisotropic crystal growth only in a structurally related MOF,^12^ although ∼10–20% lattice mismatch has been present in the formation of crystal films of multilayers of structurally related 2-dimensional MOFs.^15^,^16^ However, rapid *in situ* growth of a shell of **2** was observed on a {101[combining macron]0}face of **1** when the pure DMF surrounding the core **1** crystal was replaced by a low supersaturation growth solution of **2** as shown in Fig. 4, S11 and S12.† The difference in crystal form of a {101[combining macron]0}surface of **1** and shell **2** is highlighted in Fig. 4a and b where approximately one rectangular growth hillock dominates the image with step heights of 1.9 nm in Fig. 4a (see Fig. S13a†) that is replaced by numerous approximately rectangular growth islands covering the same area in Fig. 4b also with step heights of 1.9 nm (see Fig. S13b†). The orientation of the rectangular growth terraces in Fig. 4a and b (see also Fig. S11 and S12†) along a ) along a 〈0001〉 direction suggests epitaxial growth of 0001) along a 〈0001〉 direction suggests epitaxial growth of direction suggests epitaxial growth of **2** on **1**. Additional evidence that **2** has grown is provided by the Raman spectrum collected from a ∼858 nm diameter sampling spot size focussed on a {101[combining macron]0} and {0001} surface of the CS-LM-MOF that both show additional dabco related peaks in the range 2840–3000 cm^–1^ due to the –CH_2_– stretching vibrations that are absent in the spectrum of **1** as seen in Fig. S14.†

The formation of **2** on **1** at the interface is somewhat surprising given the lattice mismatch in the *c*-direction that will result in the first terrace of **2** containing a majority of Zn_2_ dimers that are unable to bind *via* the bdc^2–^ ligands to the underlying surface of **1** as shown in Fig. 4c. Fig. 4c represents a 3-on-2 model at the interface where three unit cells of **2** are connected to two unit cells of **1**. This 3-on-2 model for the interface appears warranted by the close agreement of the lattice parameters over these unit cell multiples, *i.e.* (9.63 Å × 3)/(14.10 Å × 2) = 1.02. The 3-on-2 matching leaves two of every three Zn_2_ dimers of **2** undercoordinated at the core–shell interface as indicated by the grey dotted lines in Fig. 4c. This exemplifies the crystal growth versatility of three-dimensional MOFs to allow continued crystal growth of infrequently attached network layers and to accommodate a high number of defects. The subsequent layers of **2** will contain a large majority of fully bdc^2–^-coordinated Zn_2_ dimers.

The crystal form of shell **2** on this facet is significantly influenced by the lattice/structure mismatching of **1** and **2** in the *ac*-plane compared to the form of **1**. The 3-on-2 model at the interface means that **2** can initially nucleate and spread at the interface through connections to **1** that occur on one of two different subsets of Zn_2_ dimer nodes at the surface of **1**. The Zn_2_ dimer nodes within either of the two subsets have a separation of 2*c*(**1**) (2 × 14.10 Å) as shown in Fig. 4c by the subset of alternate Zn_2_ dimers of **1** (yellow) that are connected to Zn_2_ dimers (blue) of **2** and those that are not. Nuclei of **2** will initially form at different Zn_2_ dimer nodes on **1**. These nuclei will then spread but their ability to coherently or incoherently converge with other spreading nuclei in both the . These nuclei will then spread but their ability to coherently or incoherently converge with other spreading nuclei in both the 〈101̄0〉 and 〈0001〉 directions will depend on their initial nucleation sites. This will create numerous growth islands that cannot simply form a continuous layer as there is extended structure incoherency at the interface of the island edges that results in line defects as shown in 101[combining macron]0. These nuclei will then spread but their ability to coherently or incoherently converge with other spreading nuclei in both the 〈101̄0〉 and 〈0001〉 directions will depend on their initial nucleation sites. This will create numerous growth islands that cannot simply form a continuous layer as there is extended structure incoherency at the interface of the island edges that results in line defects as shown in and . These nuclei will then spread but their ability to coherently or incoherently converge with other spreading nuclei in both the 〈101̄0〉 and 〈0001〉 directions will depend on their initial nucleation sites. This will create numerous growth islands that cannot simply form a continuous layer as there is extended structure incoherency at the interface of the island edges that results in line defects as shown in 0001. These nuclei will then spread but their ability to coherently or incoherently converge with other spreading nuclei in both the 〈101̄0〉 and 〈0001〉 directions will depend on their initial nucleation sites. This will create numerous growth islands that cannot simply form a continuous layer as there is extended structure incoherency at the interface of the island edges that results in line defects as shown in directions will depend on their initial nucleation sites. This will create numerous growth islands that cannot simply form a continuous layer as there is extended structure incoherency at the interface of the island edges that results in line defects as shown in Fig. 4d. These line defects will influence the mechanism and rates of terrace spreading in both the . These line defects will influence the mechanism and rates of terrace spreading in both the 〈101̄0〉 and 〈0001〉 directions in this and subsequent growth layers of 101[combining macron]0. These line defects will influence the mechanism and rates of terrace spreading in both the 〈101̄0〉 and 〈0001〉 directions in this and subsequent growth layers of and . These line defects will influence the mechanism and rates of terrace spreading in both the 〈101̄0〉 and 〈0001〉 directions in this and subsequent growth layers of 0001. These line defects will influence the mechanism and rates of terrace spreading in both the 〈101̄0〉 and 〈0001〉 directions in this and subsequent growth layers of directions in this and subsequent growth layers of **2** reducing the probability of forming large continuous growth layers such as those seen in Fig. 4a. Again the growth of **2** on the {101[combining macron]0}faces introduces undercoordinated Zn atoms at the interface of **1** and **2** and in subsequent growth terraces of **2**.

**Fig. 5:**
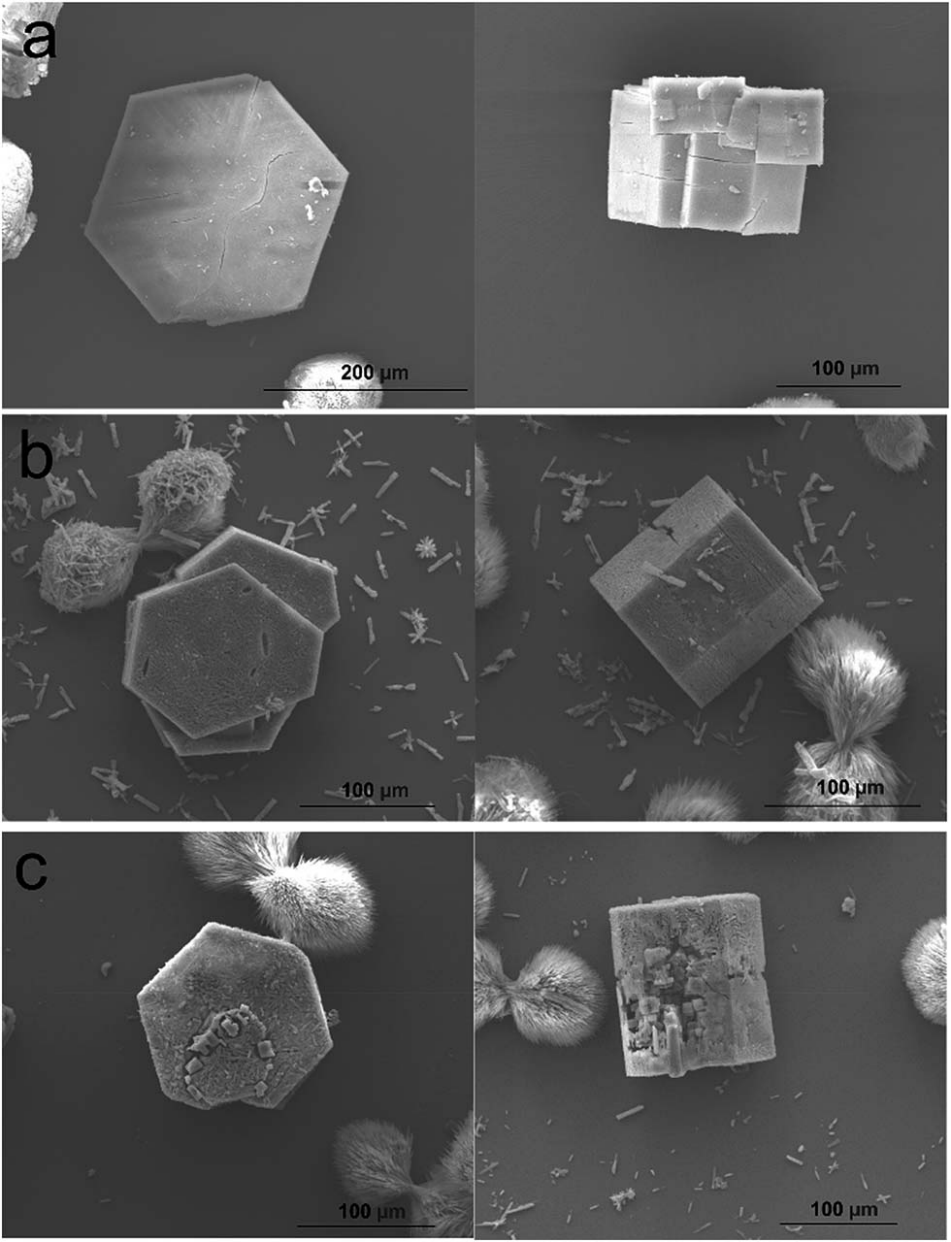
Scanning electron micrographs of hexagonal prismatic crystals of **1** (a), core shell crystals (**1**/**2**) 1 h after exposure to a growth solution of **2** (b) and core shell crystals (**1**/**2**) 24 h after exposure to a growth solution of **2** (c).

The effect on the overall crystal form of growing **2** on **1** is evidenced in the *ex situ* scanning electron micrographs (SEM) shown in Fig. 5. Hexagonal prismatic crystals of **1** with clearly expressed {0001} and {101[combining macron]0}facets are shown in Fig. 5a and S2.† The surfaces of the crystals are generally observed to be flat but contain some intergrowths. Exposure of **1** to a growth solution of **2** under conditions similar to the *in situ* AFM experiments for 1 h yields similarly well-defined crystals of CS-LM-MOF although the crystal surfaces appear slightly rougher than in **1** as shown in Fig. 5b. Exposure of **1** to a growth solution of **2** under conditions similar to the *in situ* AFM experiments for 24 h (a time considerably longer than that of the *in situ* AFM experiments) still yield hexagonal prismatic crystals of CS-LM-MOF that now show clear indications of crystal dissolution most noticeably on the {101[combining macron]0}facets as shown in Fig. 5c. This observation indicates that the CS-LM-MOF is not stable over extended periods of time under a growth solution of **2** and is most likely to undergo dissolution of domains of the core **1**. It is also evident that dissolution through the shell **2** on the {101[combining macron]0}facets is more rapid which is likely to be due to the form, and defects contained within, of **2** on these facets.

## Conclusions

*In situ* AFM has unveiled, for the first time, the route through which a coordination compound can overcome framework and crystal mismatches to grow a CS-LM-MOF and demonstrate how the form of the CS-LM-MOF is influenced by this mismatch. The shell of this CS-LM-MOF contains a much larger number of partially coordinated unsaturated metal sites than would be expected in a crystallite of the shell MOF only, thus potentially providing CS-LM-MOFs with additional properties, for example open metal adsorption sites or Lewis acid catalytic sites, than those expected for the simple combination of two MOFs in a core shell composite. Such understanding of the formation of CS-LM-MOFs will aid the future design and synthesis of such complex MOFs, with particular regard to the engineering of the interfacial defects, and lattice-mismatched epitaxially grown materials in general.

## Conflicts of interest

There are no conflicts to declare.

## Supplementary Material

Supplementary information
