# Supplementary material for: Unveiling the mechanism of lattice-mismatched crystal growth of a core–shell metal–organic framework
Source: Chem Sci. 2019 Aug 27;10(41):9571–5. doi: 10.1039/c9sc03131f (PMC6979500; doi:10.1039/c9sc03131f)
Supplement: Supplementary file 1 [file SC-010-C9SC03131F-s001.pdf]

Electronic Supplementary Material (ESI) for Chemical Science.
This journal is © The Royal Society of Chemistry 2019

Unveiling the mechanism of lattice-mismatched crystal growth of a core-shell metal-organic framework

Fajar I. Pambudi, Michael W. Anderson and Martin P. Attfield

Supporting Information

Contents:

Experimental Section:

1. Synthesis of [$Zn_2(bdc)_2(bpy)$] (**1**) and [$Zn_2(bdc)_2(dabco)$] (**2**)

2. Materials characterization

Tables:

Table S1. Summary of the in-situ AFM experiments.

Table S2. Band assignment for the Raman spectra.

Abridged figure captions:

Figure S1. Powder X-ray diffraction pattern of the crystalline product formed from the synthesis of **1** showing a mixture of the hexagonal (blue tick marks) and triclinic (red tick marks) crystalline phases.

Figure S2. Scanning electron (left) and optical (right) micrographs of crystals of **1** showing the hexagonal prism shape with clearly expressed {0001} (a) and $\{10\bar{1}0\}$ (b) facets.

Figure S3. AFM deflection images with associated cross-sectional analyses for the (0001) facet of **1** 44.6 min (a) and 46.2 min (b) after injecting a growth solution of **1**. (Table S1 Experiment 1).

Figure S4. The AFM deflection image with associated cross-sectional analyses for the $(10\bar{1}0)$ facet of **1** 25.7 min after injecting a growth solution of **1**. (Table S1 Experiment 2).

Figure S5. Structure of the of **1** (a), (b)and **2** (c) viewed along the $[01\bar{1}0]$ direction with the associated surface termination planes at X, X', X_a and X_b and the corresponding heights of the meta-stable sublayers and stable layers through which the structures are grown marked.

Figure S6. AFM deflection images with associated cross-sectional analyses taken during the in-situ AFM experiment (Table S1 Experiment 5) on the (0001) face of crystal **1** under DMF after 1.6 (a) and 3.2 (b) min from the start of scanning.

Figure S7. AFM deflection images with associated cross-sectional analyses taken during the in-situ AFM experiment (Table S1 Experiment 6) on the (0001) face of crystal **1** under DMF after 1.4 (a) and 3.4 (b) min from the start of the scanning.

Figure S8. A series of AFM deflection images (a – h) with associated cross-sectional analyses during the in-situ AFM experiment (Table S1 Experiment 4) on the (0001) face showing a d_{0001}-spacing monolayer of **2** completely overgrowing a 0.42 ± 0.1 nm high island of **2** over a period of ~8 minutes.

Figure S9. The full sequence of AFM deflection images with associated cross-sectional analyses from which Figure 3 was produced that shows a d_{0001}-spacing monolayer of **2** overgrowing a large 0.4 nm high island of **2** and growing around smaller 0.4 nm high islands of **2** (Table S1 Experiment 4).

Figure S10. An AFM deflection image (a) and its magnified image (b) showing the hexagonal growth island (green dashed line in (b)) and (c) its ~30° misalignment with respect to the bulk crystal orientation (green dashed line in (c)).

Figure S11. AFM deflection images taken over similar areas during an in-situ growth experiment (Table S1 Experiment 8) on the $(10\bar{1}0)$ facet of crystal **1** under DMF (a) and 30.6 min after after injecting the growth solution of **2** (b) highlighting the difference in surface form after growth of **2**.

Figure S12. Optical micrograph of the AFM tip together with the core crystal of **1** (a) and the AFM deflection images taken during an in-situ growth experiment (Table S1 Experiment 9) on the $(10\bar{1}0)$ facet of crystal **1** under DMF (b) and 43.2 min after injecting the growth solution of **2** (c) highlighting the difference in surface form after growth of **2**.

Figure S13. AFM deflection images with associated cross-sectional analyses for the $(10\bar{1}0)$ facet of **1** under DMF (a), and after 189 min (b) after injecting a growth solution of **2**. (Table S1 Experiment 7).

Figure S14. Raman spectra of a silicon substrate (black), bdc (pink), dabco (beige), bpy (yellow), a crystal of **2** (green), a core crystal of **1** (red), a (0001) facet of a core-shell (**1**/ **2**) (blue) and a $(10\bar{1}0)$ facet of a core-shell (**1**/ **2**) (cyan).

Experimental Section

1. Synthesis of $[Zn_2(bdc)_2(bpy)]$ (**1**) and $[Zn_2(bdc)_2(dabco)]$ (**2**)

Synthesis of $[Zn_2(bdc)_2(bpy)]$ (1). A sample of **1** were prepared according to the method by Kondo et al.[1] to form hexagonal prism-shaped crystals. A mixture of $Zn(NO_3)_2 \cdot 6H_2O$ (59.6 mg, 0.2 mmol, Sigma Aldrich 98%), H_2bdc (33.2 mg, 0.2 mmol, Sigma Aldrich 98%), and 4,4'-bipyridine (15.6 mg, 0.2 mmol, Acros Organics 98%) was added to 20 mL of N,N'-dimethylformamide (DMF, Sigma Aldrich ≥99.8%) in a 43 mL glass jar. The mixture was sonicated for 30 min to obtain a clear solution. A round borosilicate glass cover slide (diameter 19 mm, thickness No. 1, VWR) was inserted into the solution. The glass jar was then tightly capped and heated in an oil bath at 120 ^oC for 60 min resulting in a clear solution. The glass jar was removed from the oil bath and allowed to cool in air to room temperature before being left for 24 hr at room temperatue. Hexagonal prism-shaped crystals of **1** crystallized on the sides of the glass jar and on the glass cover slide during the period at room temperature. In addition to **1**, another crystalline triclinic phase of different crystal morphology was formed as observed in the powder XRD pattern (Figure S2) and reported previously.[1] The remaining reaction solution was used directly as the supernatant/ growth solution of **1** for the subsequent in situ AFM experiments. The aging time of 1 day was selected as the supersaturation was low enough to observe individual surface nucleations in the in situ AFM experiments.

Synthesis of $[Zn_2(bdc)_2(dabco)]$ (2). A sample of **2** was prepared to produce the growth solution **2** for the in-situ AFM experiments following the method of Chun et al.[2] A mixture of $Zn(NO_3)_2 \cdot 6H_2O$ (59.6 mg, 0.2 mmol, Sigma Aldrich 98%) and H_2bdc (33.2 mg, 0.2 mmol, Sigma Aldrich 98%) was dissolved in 2.5 mL of a DMF/CH_3CN (DMF, Sigma Aldrich ≥99.8%; CH_3CN, Fisher Chemical, HPLC grade) mixture with a ratio of 4:1 (v/v) by sonicating for 15 min. 1,4-diazabicyclo[2.2.2]octane (15 mg, 0.13 mmol, Sigma Aldrich ≥99%) was added to the clear solution that was further sonicated for 30 min to produce a turbid solution. The mixture was then centrifuged at 3000 rpm to separate any undissolved reagent. The clear solution was placed in a 15 mL glass vial and anhydrous ethylene glycol (0.7 mL, Sigma Aldrich 99.8%) was added. The glass vial was tightly capped and heated in an oven at 120 ^oC for 2 days before being

removed and air cooled to room temperature to yield hexagonal prism-shaped crystals. The glass vial was left at room temperature for an additional 13 days and then the mother liquor (supernatant) was decanted from the resulting crystalline product. The aging time of 13 days was selected as the supersaturation was low enough to observe individual surface nucleations in the in situ AFM experiments which was not possible when the supernatant was aged for shorter periods at room temperature as the growth of layers of **2** was very rapid. The zinc concentration in this solution was 140 ppm compared to approximately 4086 ppm in the initial synthesis mixture, 186 ppm after the synthesis mixture was heated at 120 ^{o}C for 2 days and 155 ppm after the synthesis mixture was heated at 120 ^{o}C and aged for 8 days. This supernatant was used as the growth solution for the in situ AFM experiments to grow the shell MOF **2**. The crystals of **2** were also separated and use in the Raman experiments.

2. Materials characterization

Powder X-ray diffraction (PXRD). PXRD was used to determine the identity and phase purity of the crystalline products. Diffraction data were collected using Philips X'pert diffractometer at ambient conditions in the 2θ range 3.5-40° using Cu-Kα radiation. The materials were loaded onto a cut silicon sample holder.

In-situ Atomic Force Microscopy. The round glass cover slide with crystals of **1** attached was removed from the remaining supernatant synthesis mixture and dipped several times in DMF to remove any remaining debris from the crystal surfaces. The cover slide with crystals of **1** attached was glued onto a rectangular glass slide upon which a home-made in-situ AFM fluid-cell was glued. The fluid-cell was made of a rectangular metal walled unit containing an inlet and outlet tube. The fluid cell was mounted on a NanoWizard II JPK Instrument AG.

A growth solution of **1** was placed in a 3 mL syringe that was attached to a syringe pump. Initially 0.7 mL of DMF was added to fill the AFM fluid-cell and AFM scanning was commenced. Subsequently a further 0.2 mL of growth solution was injected into the fluid cell using the automatic syringe pump at a flow rate of 0.05 mL min^{-1}. The injection rate of the growth solution was kept as low as possible to minimize any disturbance during scanning. The crystal surface was scanned continuously under this initially dynamic, and then static, growth solution throughout this procedure to try

to capture the entire crystal growth process. In-situ AFM experiements using growth solutions of the shell MOF **2** were performed following the same procedure.

All in-situ AFM experiments were performed on selected hexagonal prismatic crystals of **1** attached to the cover slide with their {0001} or $\{10\bar{1}0\}$ facets approximately parallel to the slide to avoid artefacts created by the non-linear displacement of the piezoelectric scanner along the z-axis. All experiments were carried out in contact mode using silicon triangular-shaped cantilevers with a nominal tip radius of curvature of 10 nm and a force constant of 0.06 N m^{-1} (DNP-10, Bruker) and a scan rate of 3.0 Hz. AFM image analysis was carried out using the JPK image processing software provided by JPK Instrument AG. The height images were used to determine the height of any nuclei and terrace layers. The height images were flattened through application of a line-by-line fitting routine followed by a plane fit to further correct for any residual tilt prior to height measurements. The height of the nuclei and terrace layers was measured using cross-section analysis with single scan lines. Single scan lines passing through the highest point of a nuclei were used to determine the heights of the nuclei.

A list of the in-situ AFM experiments presented in this work is provided in **Table S1**.

Table S1. Summary of the in-situ AFM experiments.

Experiment number	Figure index and crystallographic face scanned	Growth solution
1	(Fig. S3) (0001)	Growth solution **1**
2	(Fig. S4) $(10\bar{1}0)$	
3	(Fig. 2) (0001)	Growth solution **2**
4	(Fig. 3, S8 - S10) (0001)	
5	(Fig. S6) (0001)	
6	(Fig. S7) (0001)	
7	(Fig. 4, S13) $(10\bar{1}0)$	
8	(Fig. S11) $(10\bar{1}0)$	
9	(Fig. S12) $(10\bar{1}0)$	

Scanning electron microscopy (SEM). SEM was used to investigate the crystal habit of crystals **1** and crystals of the core-shell **1/ 2**. The crystal images were collected using

a Quanta environmental scanning electron microscope (20 kV, 90 - 110 μA). A sample of crystals of **1** were prepared in the same manner as that described above except that two circular borosilicate glass cover slides were placed in the glass jar. After the reaction was complete, a sample of crystals was removed from the glass jar wall and dried before SEM characterisation (core crystals of **1**). One of the cover slides with attached crystals of **1** was treated and attached to a home-made in-situ AFM fluid-cell as described above. 0.7 mL DMF was added to the fluid cell followed by injection of 0.2 mL of a growth solution of **2**. This sample was kept under this static growth solution for 1 hr before the fluid cell was drained and the core-shell **1**/ **2** crystals dried in air prior to SEM characterisation. The other cover slide was subjected to the same protocol except that it was left under the static growth solution for 24 hr.

Optical microscopy. Optical micrographs of crystals were taken using Zeiss Axiovert 200 atached to the AFM NanoWizard II JPK Instrument AG. The pictures were taken during in-situ AFM experiments and show the crystals of **1** together with the AFM cantilever.

Raman spectroscopy. Core and core-shell crystals were analysed using a Renishaw Invia Raman Spectrometer using excitation radiation of wavelength of 633 nm and a laser spot size of diameter ~858 nm. A silicon wafer was used as the sample substrate during all the measurements. The {0001} and $\{10\bar{1}0\}$ facets of a number of hexagonal prismatic crystals were analysed to identify the functional groups present in pure core **1** crystals, pure shell **2** crystals and core-shell crystals **1**/ **2**. The Raman spectra were analysed by comparing the spectra of the three samples to ascertain whether **2** had grown on all facets of **1** in the core-shell crystal **1**/ **2**.

The core-shell crystals of **1**/ **2** was synthesised in the same way as that described above for the SEM sample that was was kept under the static growth growth solution of **2** for 1 hr. After exposure to the growth solution, the resulting core-shell crystals **1**/ **2** were collected from the growth solution and washed with DMF before drying in air.

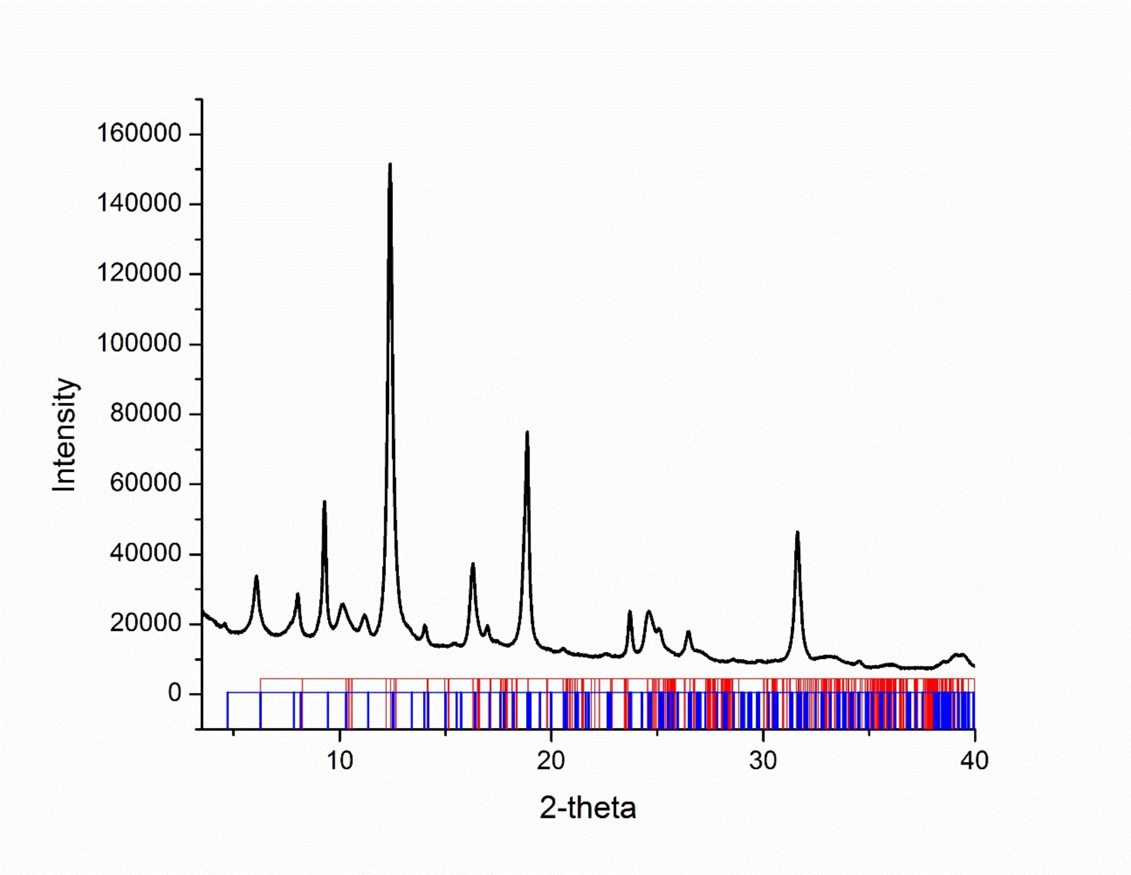

Figure S1. Powder X-ray diffraction pattern of the crystalline product formed from the synthesis of **1** showing a mixture of the hexagonal (blue tick marks) and triclinic (red tick marks) crystalline phases. The experimental diffraction pattern is shown as the black line, and the blue and red tick marks represent the calculated reflection positions for **1** (hexagonal, P6/mmm, a = 21.619(8) Å, c = 14.104(5) Å, α = 90 °, β = 90 °, γ = 120 °) and the triclinic phase ($P\bar{1}$, a = 10.886(3) Å, b = 10.919(3) Å, c = 14.091(3) Å, α = 89.294(4) °, β = 89.081(4) °, γ = 79.691(3) °)[1] respectively.

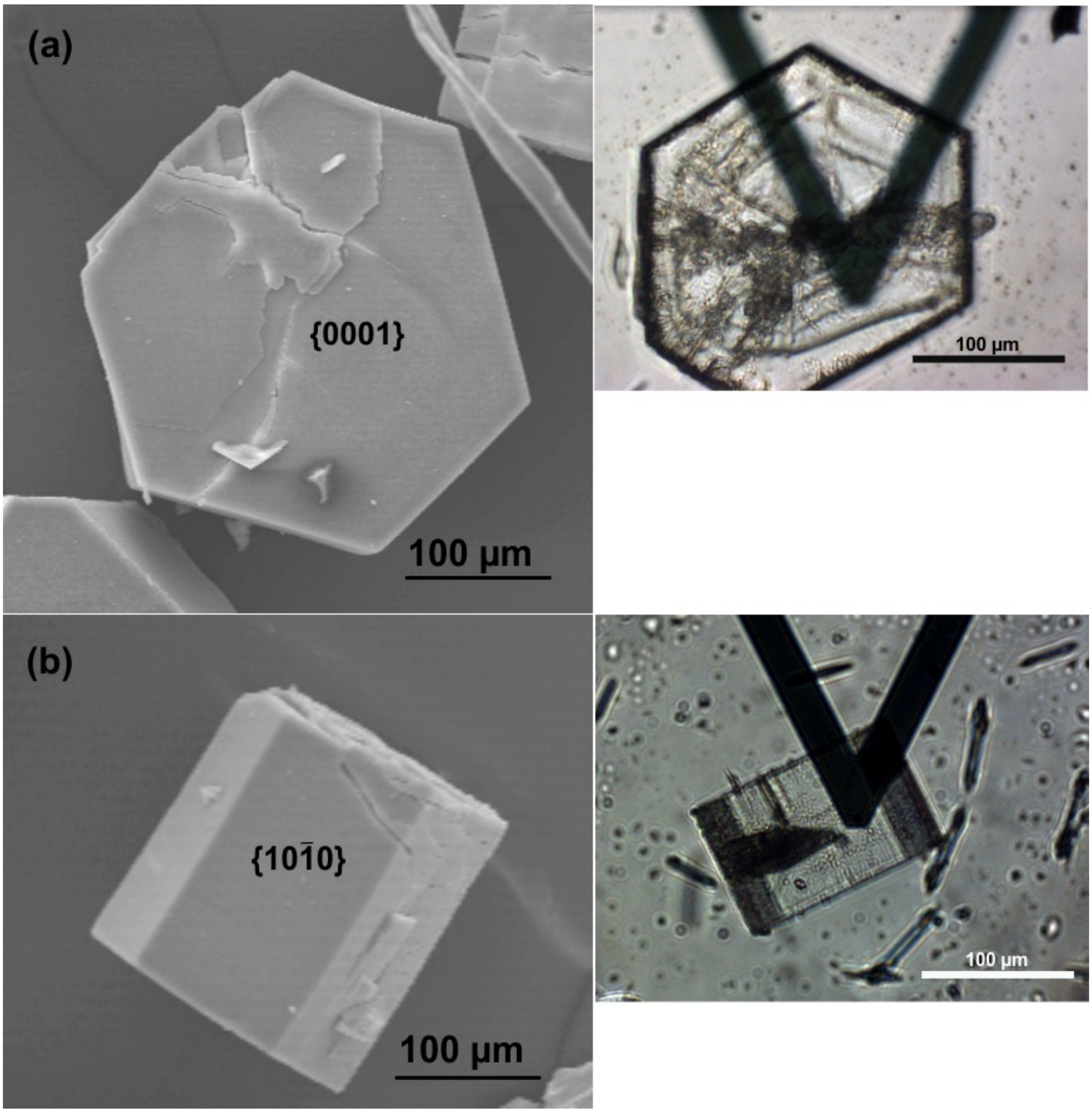

Figure S2. Scanning electron (left) and optical (right) micrographs of crystals of **1** showing the hexagonal prism shape with clearly expressed {0001} (a) and $\{10\bar{1}0\}$ (b) facets. The AFM tips are also visible in the optical micrographs.

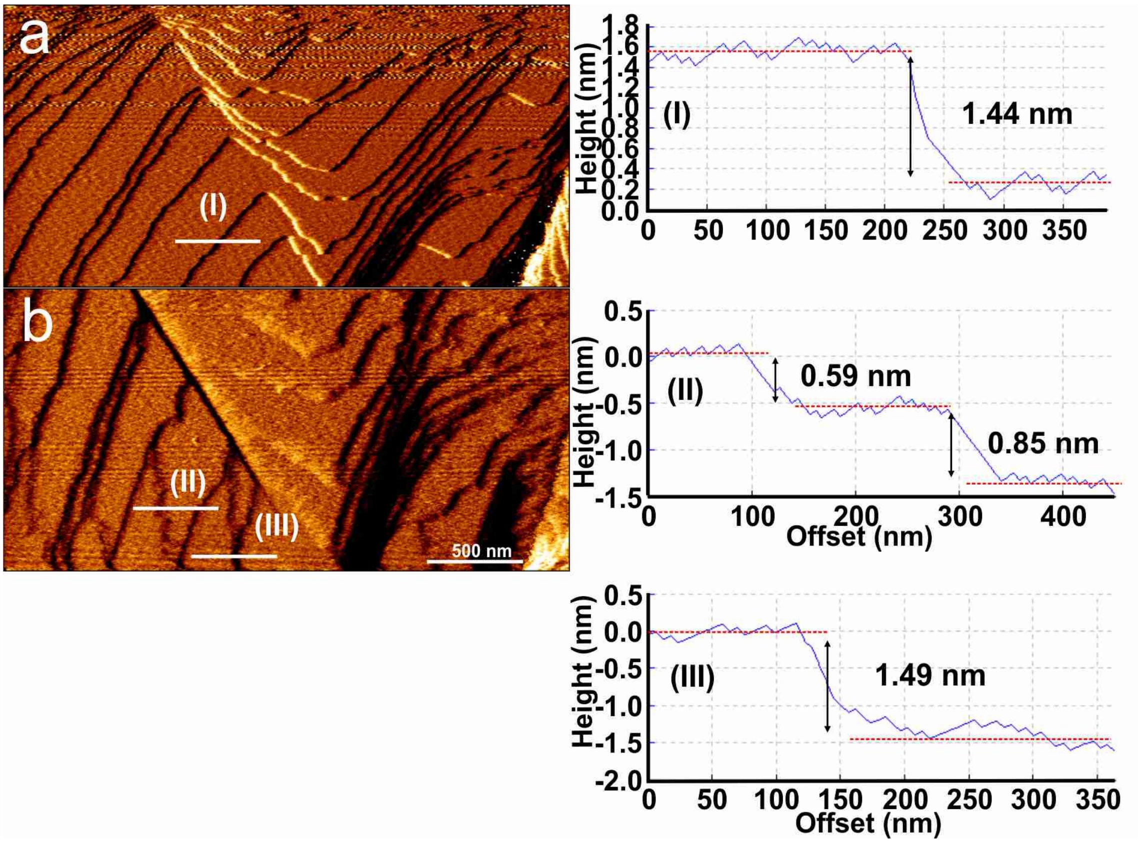

Figure S3. AFM deflection images with associated cross-sectional analyses for the (0001) facet of **1** 44.6 min (a) and 46.2 min (b) after injecting a growth solution of **1**. (Table S1 Experiment 1). The cross-sectional analyses reveal two distinct step heights in (b) II (0.59 nm and 0.85 ± 0.1 nm) corresponding the height of a [$Zn_2(bdc)_2$] layer and a bpy ligand respectively (see Figure S5b). AFM image size is 3 x 1.5 μm^2.

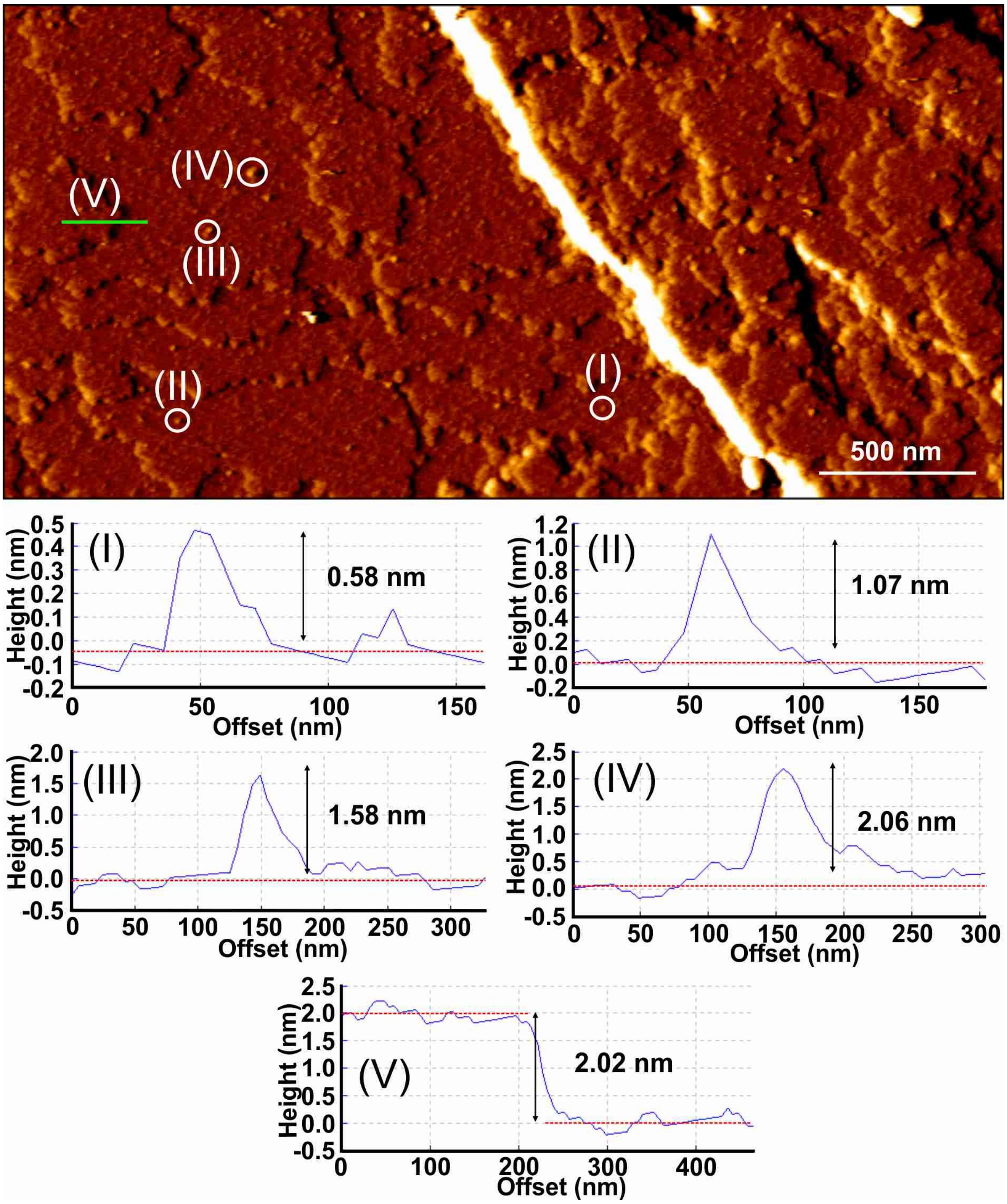

Figure S4. The AFM deflection image with associated cross-sectional analyses for the $(10\bar{1}0)$ facet of **1** 25.7 min after injecting a growth solution of **1**. (Table S1 Experiment 2). The cross-sectional analyses of several nuclei are observed with heights of 0.58, 1.07, 1.58 and 2.06 ± 0.1 nm providing some information on the growth process of the 2D nuclei on this facet and, through comparison with the atomic distances derived from the crystal structure, the possilbe the terminating surfaces of the $\{10\bar{1}0\}$ facets (see Figure S5a). AFM image size is 3 x 1.5 μm^2.

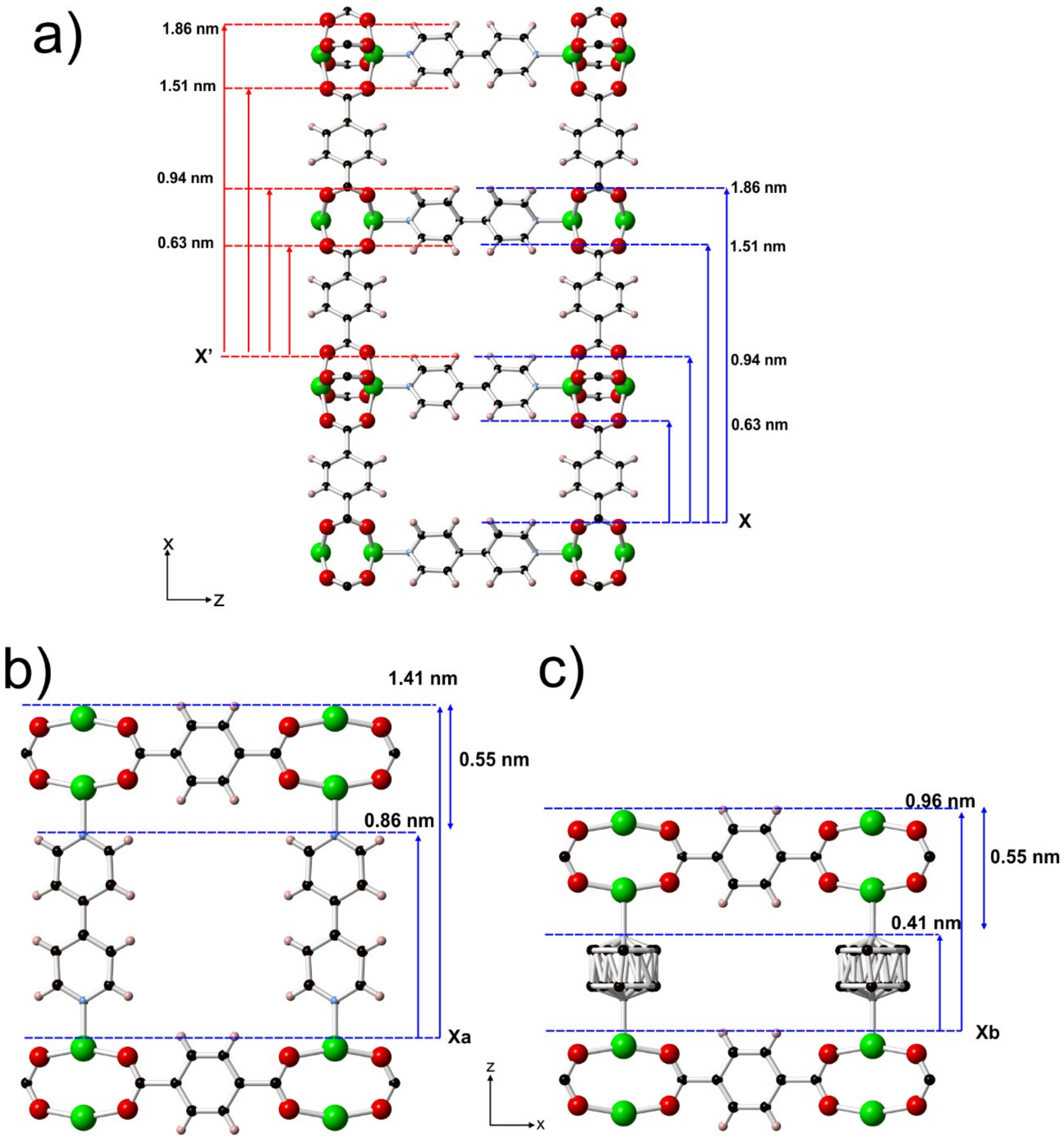

Figure S5. Structure of the of **1** (a), (b)and **2** (c) viewed along the $[01\bar{1}0]$ direction with the associated surface termination planes at X, X', X_a and X_b and the corresponding heights of the meta-stable sublayers and stable layers through which the structures are grown marked. The structures are represented in ball-and-stick mode: green: Zn, red: O, light blue: N, black: C, pink: H.

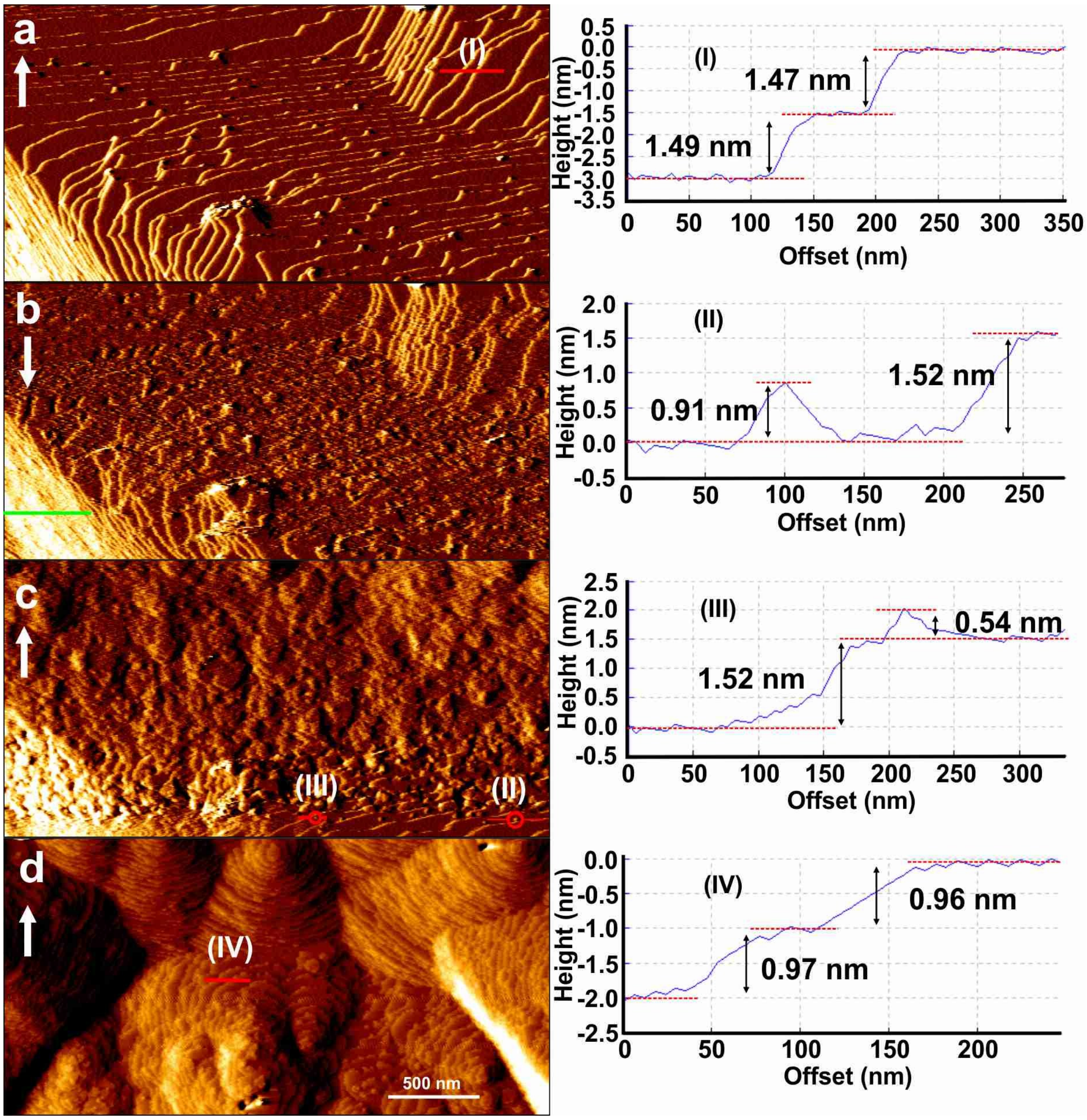

Figure S6. AFM deflection images with associated cross-sectional analyses taken during the in-situ AFM experiment (Table S1 Experiment 5) on the (0001) face of crystal **1** under DMF after 1.6 (a) and 3.2 (b) min from the start of scanning showing the terraced surface with a monolayer step height of 1.5 ± 0.1 nm (I). Surface dissolution occurred during scanning as seen by contrasting (a) and (b). The scanning was continued during injection of the growth solution of **2** towards the end of image (b) (at the point indicated by green line in (b)). The top of the subsequent image at 4.6 min (c) shows many new growth islands and terraces of **2** but at the bottom of image (c) several 2D nuclei can be clearly observed growing on the terraces of **1**. Cross-section analyses on these 2D nuclei indicate the step height of 0.9 nm (II) relating to the d_{0001} spacing of **2**. In addition, the observation of the height of 0.5 nm on (III), and comparison with the

atomic distances derived from the crystal structure (see Figure S5c), suggests the initial formation of a 2D nuclei by attachment of a dabco ligand to the crystal surface of **1**. After 7.8 min (d), the surface of **1** is completely covered with many layers of **2** of terrace height of 1.0 nm (IV). The scan direction in (a) – (d) is indicated by the white arrow. AFM image size is 3 x 1.5 μm^2.

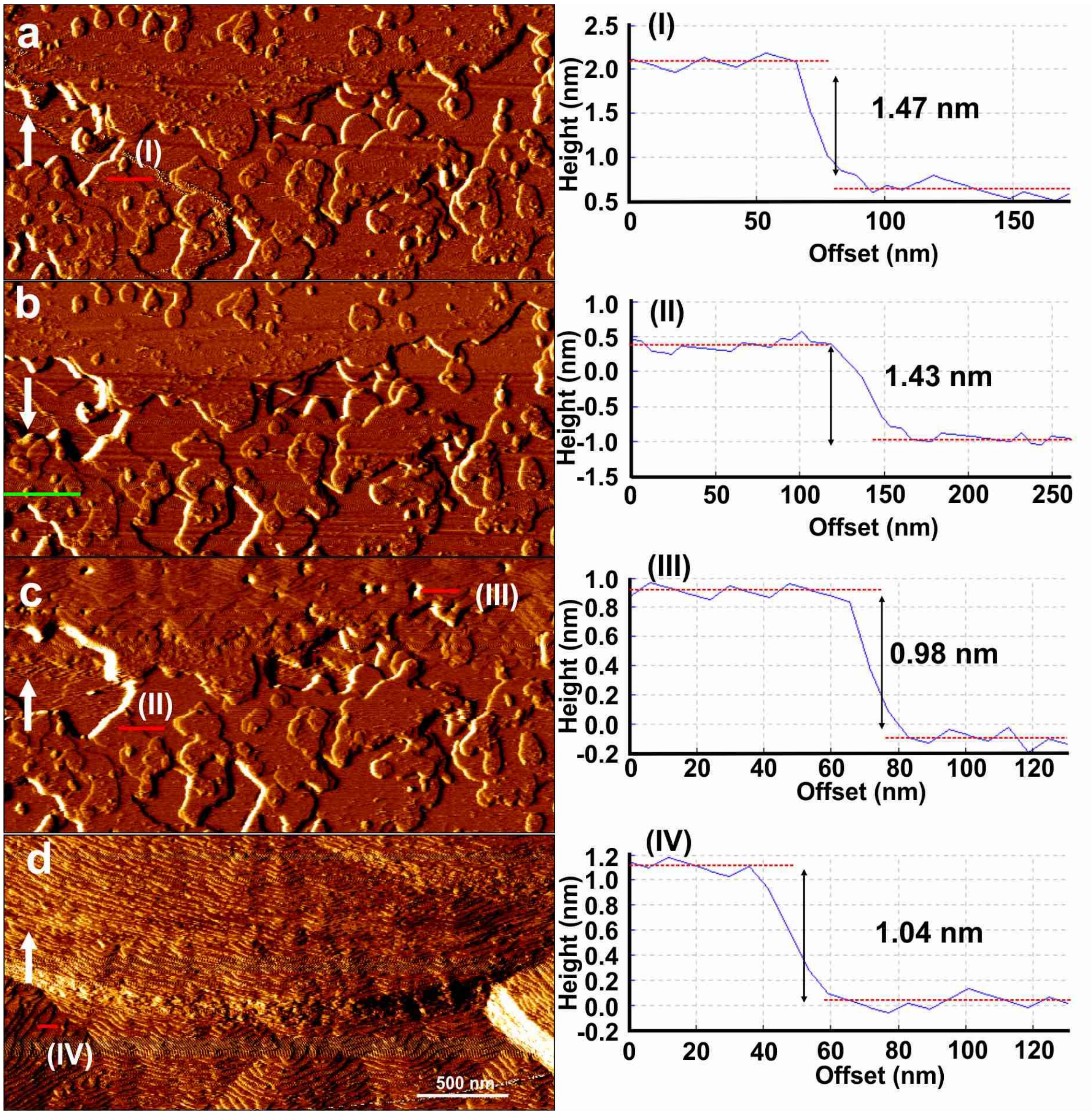

Figure S7. AFM deflection images with associated cross-sectional analyses taken during the in-situ AFM experiment (Table S1 Experiment 6) on the (0001) face of crystal **1** under DMF after 1.4 (a) and 3.4 (b) min from the start of the scanning showing the terraced surface with a monolayer step heights of 1.5 ± 0.1 nm (I) corresponding to the d_{0001} spacing of crystal **1**. The growth solution of crystal **2** was injected towards the end of the scanning process of image (b) at the point indicated by the green line. The image taken after 5.1 min (c) displays the presence of monolayer high growth layers corresponding to **1** (II) and **2** (III) at the bottom and top of the image respectively. After 8.5 min (d) of scanning the crystal surface is covered with growth layers of **2** with a representative step height of 1.0 nm (IV). The scan direction in (a) – (d) is indicated by the white arrow. AFM image size is 3 x 1.5 µm^2.

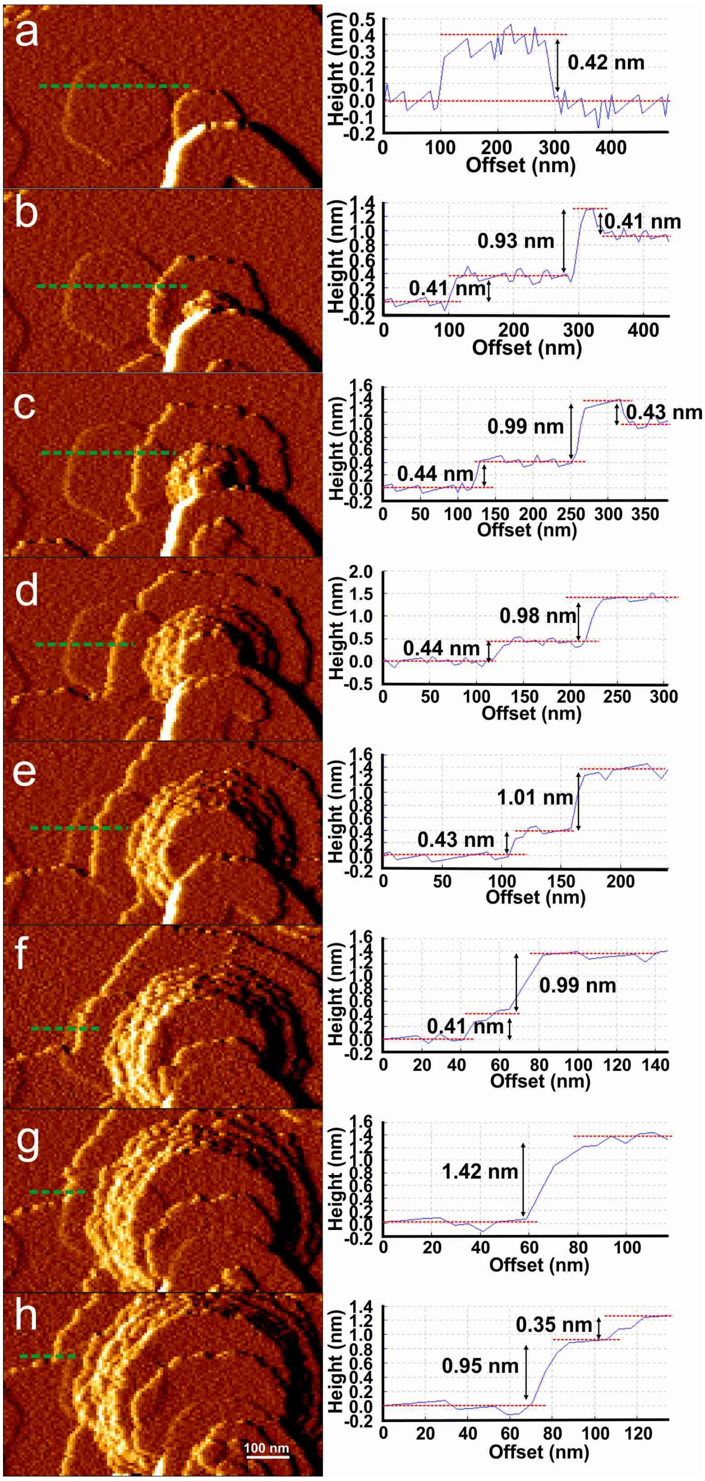

Figure S8. A series of AFM deflection images (a – h) with associated cross-sectional analyses during the in-situ AFM experiment (Table S1 Experiment 4) on the (0001) face showing a d_{0001}-spacing monolayer of **2** completely overgrowing a 0.4 ± 0.1 nm high island of **2** over a period of ~8 minutes. The 0.4 nm high heaxagonal feature seen in (a) is still oberservable in (h) after it has been covered by many layers of **2**. AFM image size is 0.7 x 0.4 μm^2.

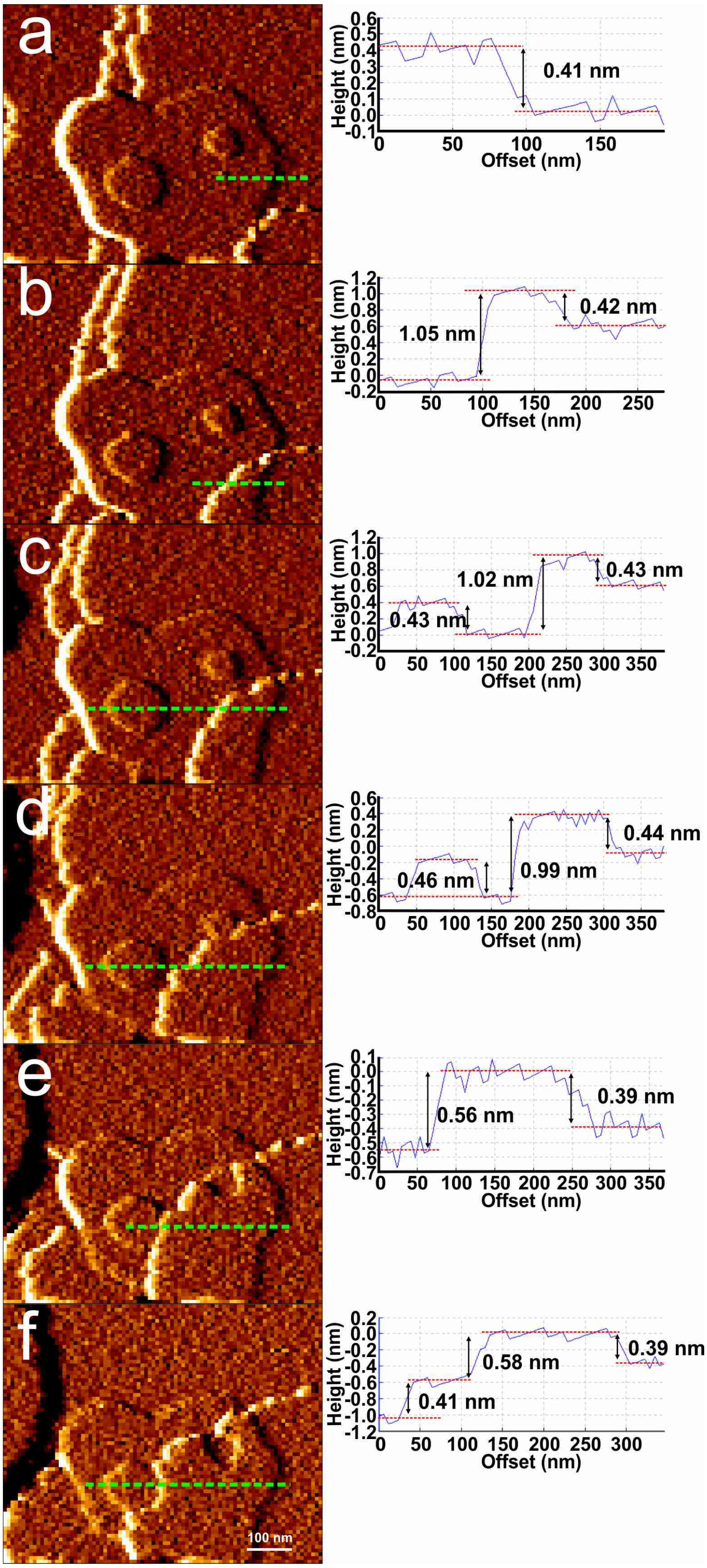
a
b
c
d
e
f
100 nm
0.41 nm
1.05 nm
0.42 nm
1.02 nm
0.43 nm
0.43 nm
0.46 nm
0.99 nm
0.44 nm
0.56 nm
0.39 nm
0.41 nm
0.58 nm
0.39 nm
Height (nm)
Offset (nm)

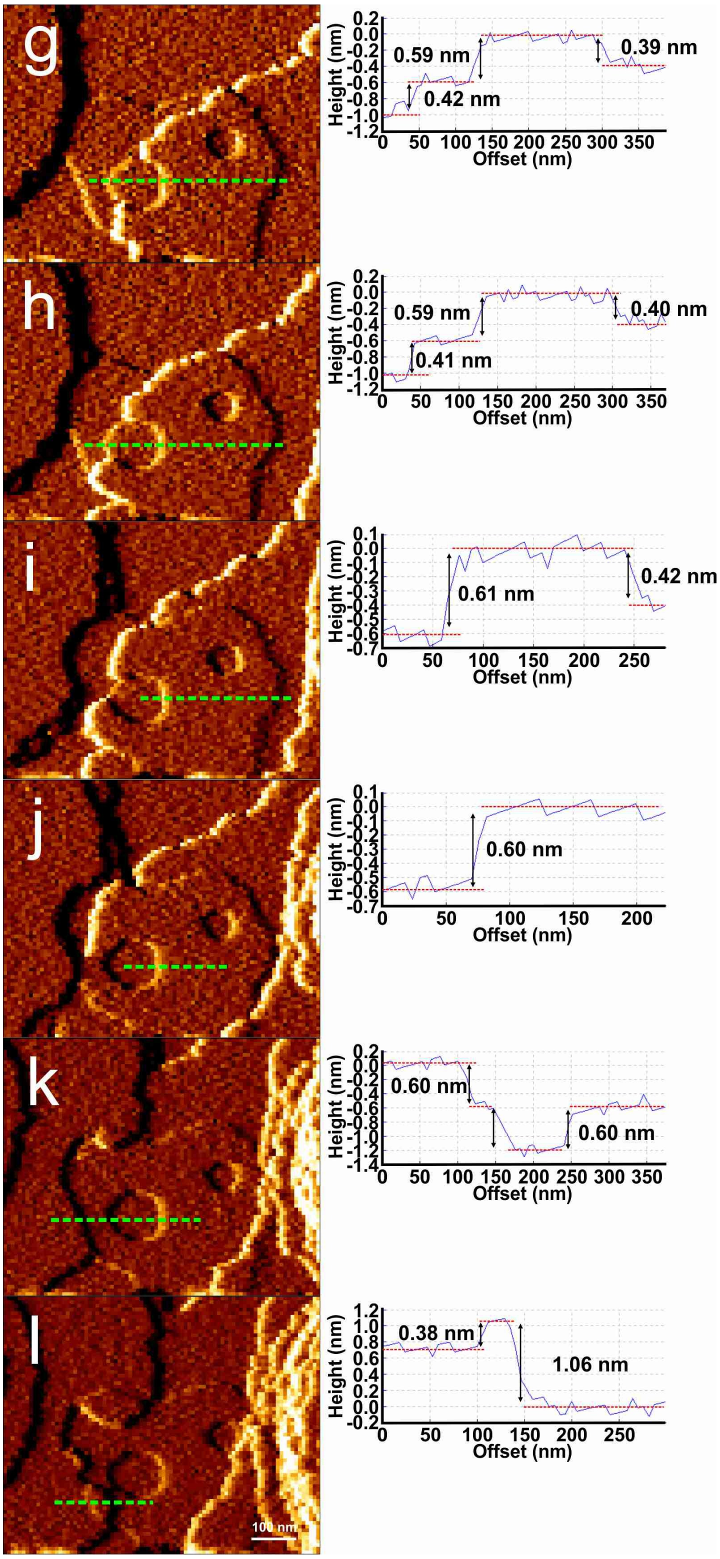

Figure S9. The full sequence of AFM deflection images with associated cross-sectional analyses from which Figure 3 was produced that shows a d_{0001}-spacing monolayer of **2** overgrowing a large 0.4 nm high island of **2** and growing around smaller 0.4 nm high islands of **2** (Table S1 Experiment 4). AFM image size is 0.7 x 0.4 µm^2.

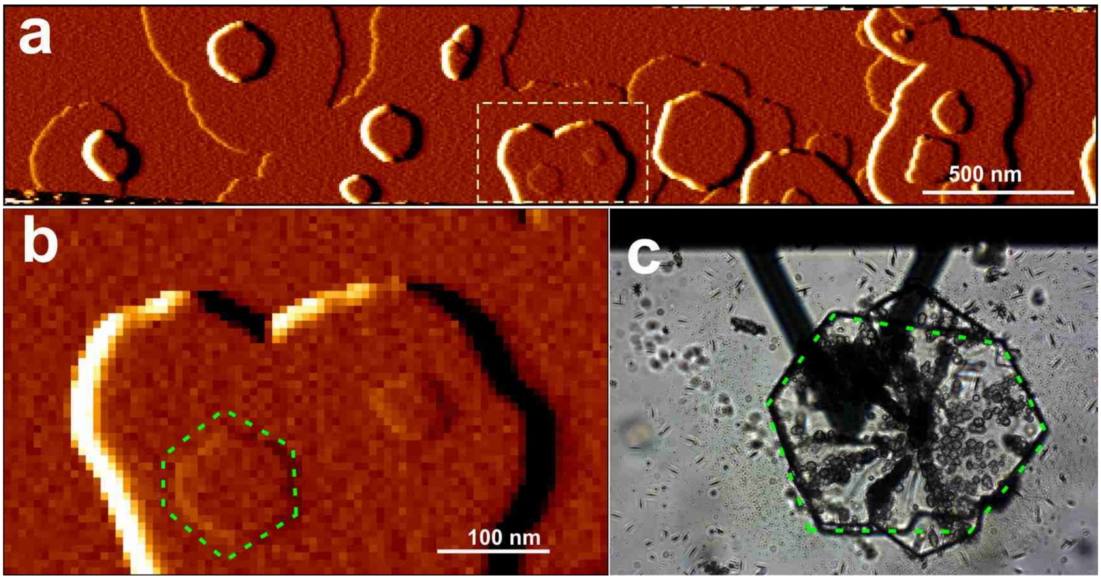

Figure S10. An AFM deflection image (a) and its magnified image (b) showing the hexagonal growth island (green dashed line in (b)) and (c) its ~30° misalignment with respect to the bulk crystal orientation (green dashed line in (c)). The AFM tip is also visible in (c).

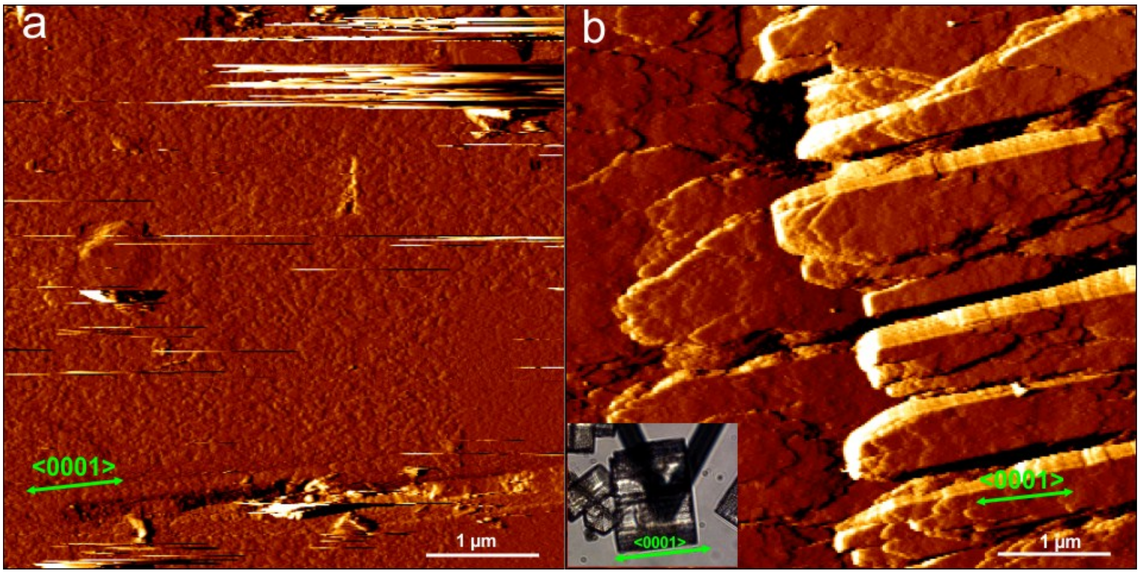

Figure S11. AFM deflection images taken over similar areas during an in-situ growth experiment (Table S1 Experiment 8) on the ($10\bar{1}0$) facet of crystal **1** under DMF (a) and 30.6 min after after injecting the growth solution of **2** (b) highlighting the difference in surface form after growth of **2**. The optical micrograph of the AFM tip together with the crystal is shown in the inset of (b). The AFM image size is 5 x 5 µm^2.

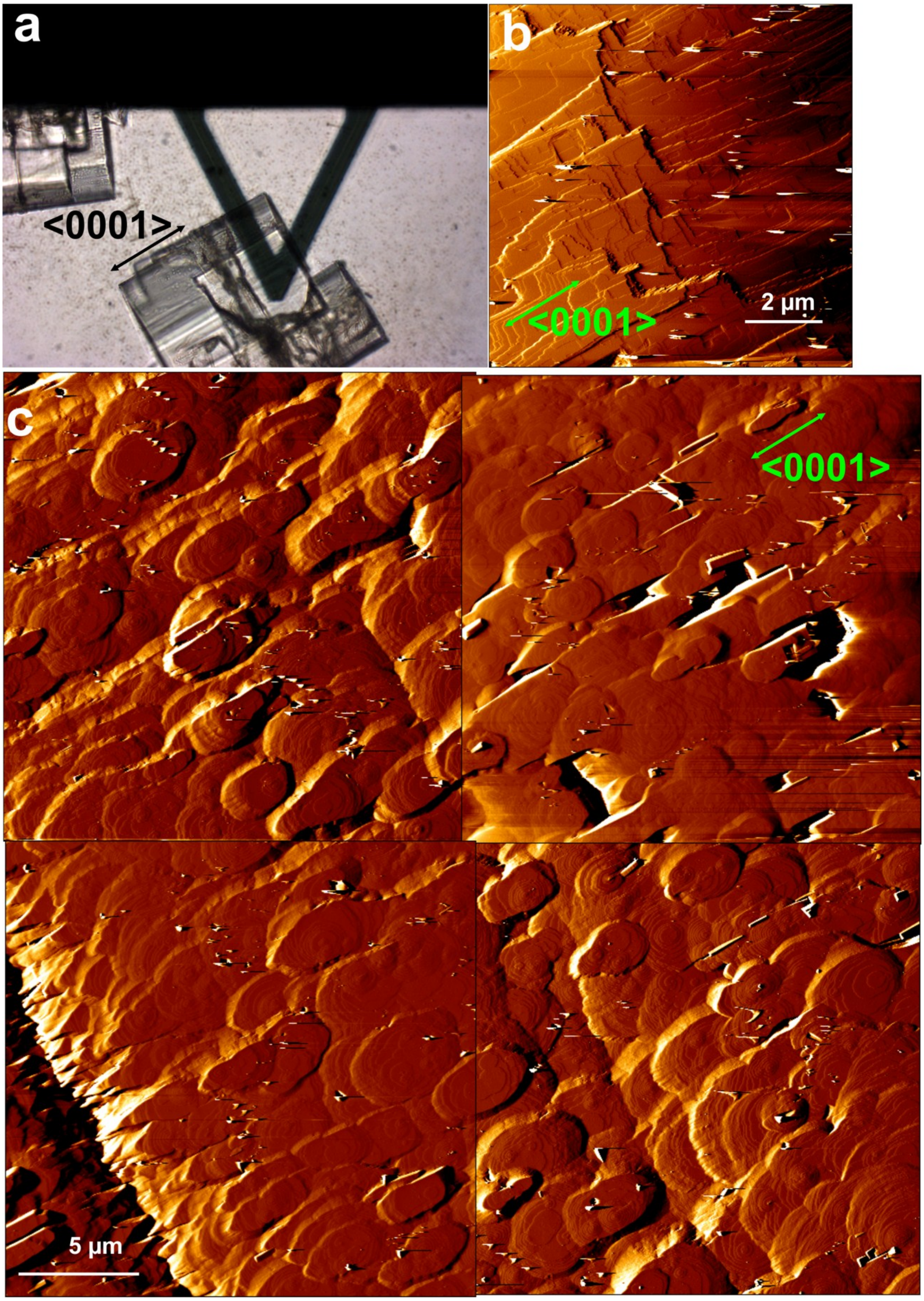

Figure S12. Optical micrograph of the AFM tip together with the core crystal of **1** (a) and the AFM deflection images taken during an in-situ growth experiment (Table S1

Experiment 9) on the ($10\bar{1}0$) facet of crystal **1** under DMF (b) and 43.2 min after injecting the growth solution of **2** (c) highlighting the difference in surface form after growth of **2**. The AFM image sizes are 10 x 10 μm^2 (b) and 40 x 40 μm^2 (c) with the area shown in (b) being contained in (c).

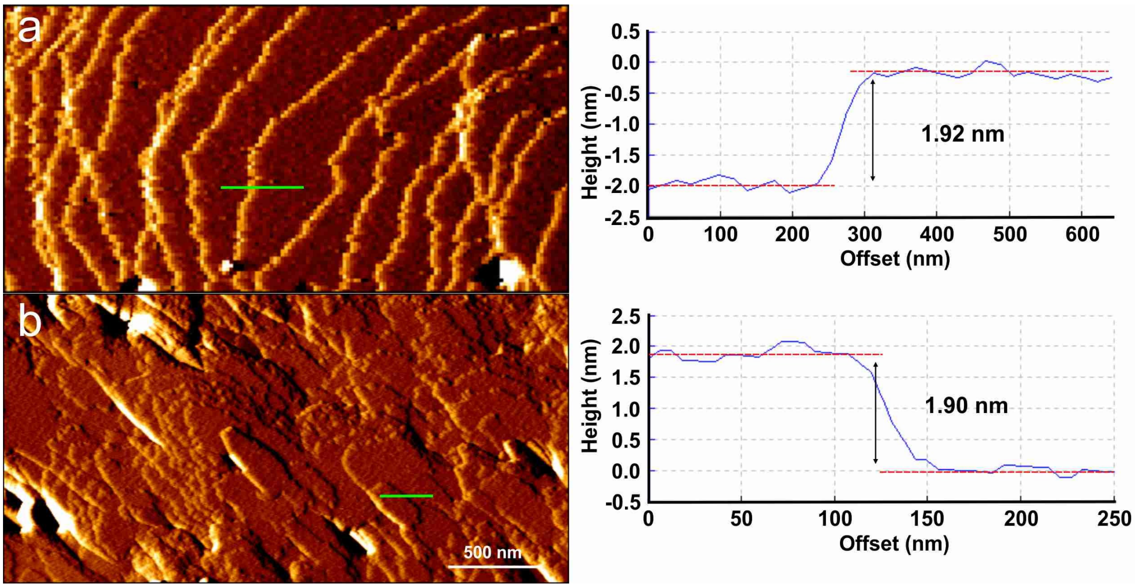

Figure S13. AFM deflection images with associated cross-sectional analyses for the ($10\bar{1}0$) facet of **1** under DMF (a), and after 189 min (b) after injecting a growth solution of **2**. (Table S1 Experiment 7).

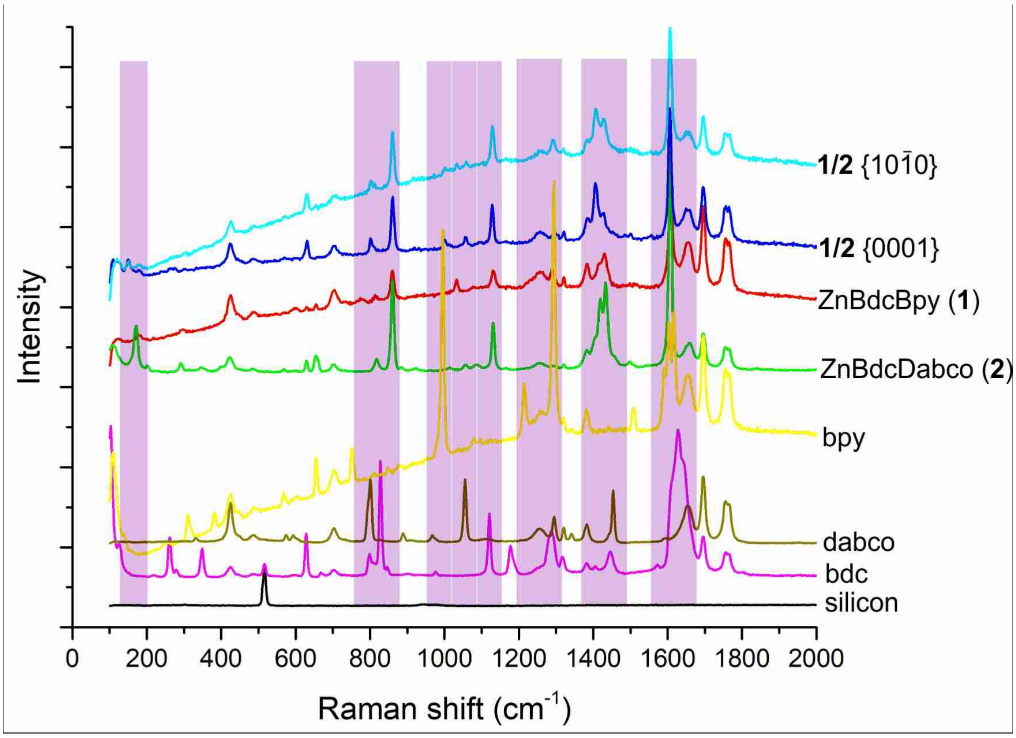

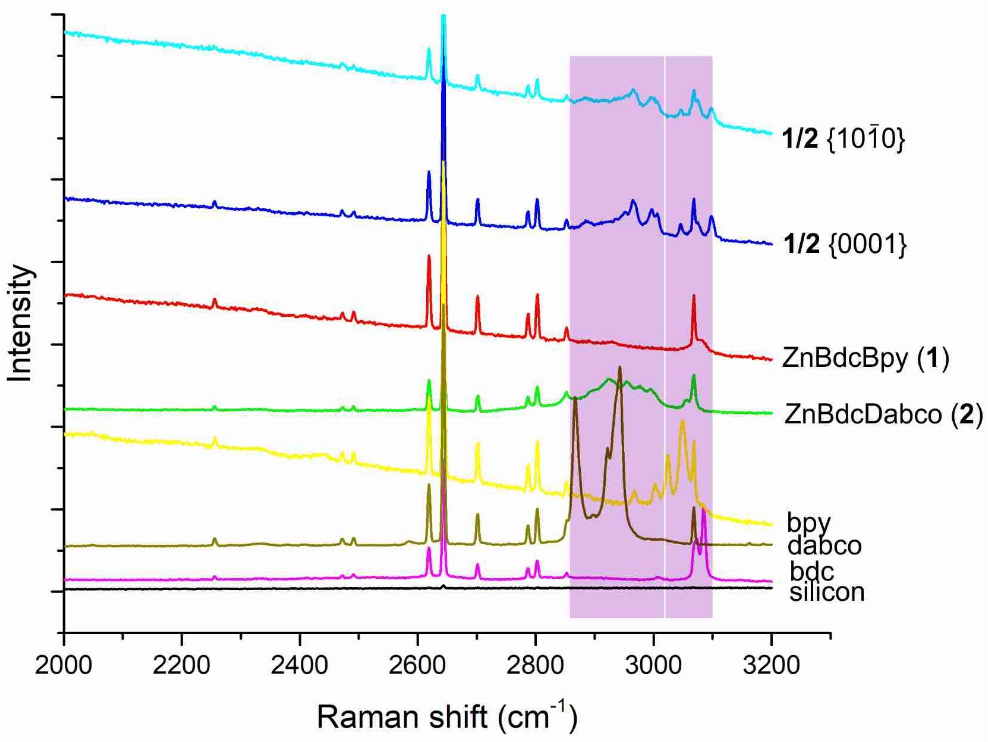

Figure S14. Raman spectra of a silicon substrate (black), bdc (pink), dabco (beige), bpy (yellow), a crystal of **2** (green), a core crystal of **1** (red), a (0001) facet of a core-shell (**1**/ **2**) (blue) and a ($10\bar{1}0$) facet of a core-shell (**1**/ **2**) (cyan).

Table S2. Band assignment for the Raman spectra.

Region peaks/ cm^{-1}	Band assignments
~3050 - 3100	ν C-H (bdc, bpy)
~2840 - 3000	ν_s –CH_2- (dabco)
~1600 - 1700	Aromatic ring mode<br>ν_s C-C (bdc), ν_s C-N, ν_{as} O-C-O<br>ring breathing mode (bdc)
~1400-1457	δ –CH_2- (dabco)<br>ν_s O-C-O
~1200-1300	δ C-H (bpy)<br>ν C-C inter ring
~1100	δ C-H (bdc)
~1050	ν C-C (dabco)
~1000	Ring breathing mode (bpy)
~861	γ C-H (bpy),
~806	ν_s NC_3
~174	ν Zn-N, ν Zn-O

ν = stretching; γ = out of plane bend; δ = in plane bend; s = symmetric; as = asymmetric [3,4,5,6]

References

[1] M. Kondo, Y. Takashima, J. Seo, S. Kitagawa, S. Furukawa, *CrystEngComm,* **2010**, 12, 2350 – 2353.

[2] H. Chun, J. Moon, *Inorg. Chem*. **2007**, 46, 4371 – 4373.

[3] H. Kihara, Y. Gondo, *J. Raman Spectrosc.* **1986**, 17, 263 – 267.

[4] D. A. Guzonas, D. E. Irish, *Can. J. Chem.* **1988**, 66, 1249 – 1257.

[5] G. Kumari, N. R. Patil, V. S. Bhadram, R. Haldar, S. Bonakala, T. K. Maji, C. Narayana, *J. Raman Spectrosc*. **2016**, 47, 149 – 155.

[6] J. Boissonnault, A. G. Wong-Foy, A. J. Matzger, *J. Am. Chem. Soc*. **2017**, 139, 14841 – 14844.
